# An Atomic‐Level Bimetallic MOF Platform Overcoming the Stability‐Performance Tradeoff for Laser Propulsion

**DOI:** 10.1002/adma.72795

**Published:** 2026-03-13

**Authors:** Senlin Rao, Gang Tang, Shizhuo Zhang, Gary J. Cheng

**Affiliations:** ^1^ Jiangxi Provincial Key Laboratory of Precision Drive and Equipment Jiangxi University of Water Resources and Electric Power Nanchang P. R. China; ^2^ The Institute of Technological Sciences Wuhan University Wuhan P. R. China; ^3^ School of Industrial Engineering Purdue University West Lafayette INDIANA USA

## Abstract

This work establishes a materials design paradigm that achieves simultaneous enhanced stability and performance for photon‐based propulsion. We introduce an atomic‐level bimetallic platform to overcome the inherent trade‐off between high thrust efficiency and environmental stability, particularly against hydrolysis. This is achieved through bimetallic FeCu‐MOFs synthesized via a one‐step laser synthesis, where Fe^3+^ and Cu^2+^ co‐crystallize with tricarboxylate ligands to form an isomorphous HKUST‐1 derivative. This approach exploits hard‐soft acid‐base principles to achieve several fundamental advances: enhanced bond strength, hydrolytic and thermal stability through the formation of robust Fe‐O bonds, increasing water resistance by 20 times while preserving crystalline integrity; synergistic, delocalized energy dissipation via d‐orbital charge transfer (Fe^3+^→Cu^2+^), boosting uniform photothermal conversion to 91%; and inherent stoichiometric tunability, where the Fe:Cu ratio serves as a precise performance lever, providing a design strategy to optimize stability and performance. The optimized FeCu‐MOF‐M variant achieves record propulsion metrics—impulse coupling coefficient (191.80 µN/W), specific impulse (631.19 s), thrust density (61.86 µN/µg), and ablation efficiency (59.32%) —surpassing monometallic HKUST‐1 by 15.7% and physical mixtures by 125%. By unifying hydrolysis resistance, efficient photothermal conversion, and atomic‐level tunability, this stoichiometry‐driven photothermal synergy bimetallic frameworks provides a solid foundation for next‐generation energetic materials in demanding environments.

## Graphical abstract

This work introduces an atomic‐level engineered bimetallic MOF Platform that resolves the inherent trade‐off between environmental stability and propulsion efficiency. By integrating robust Fe‐O bonds for hydrolysis resistance and enabling d‐orbital charge transfer (Fe^3+^→Cu^2+^) for enhanced photothermal conversion, the design achieves a highly durable, record‐breaking propellant for laser‐based space exploration.

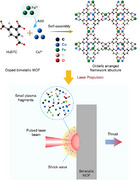

## 1 Introduction

**FIGURE 1:**
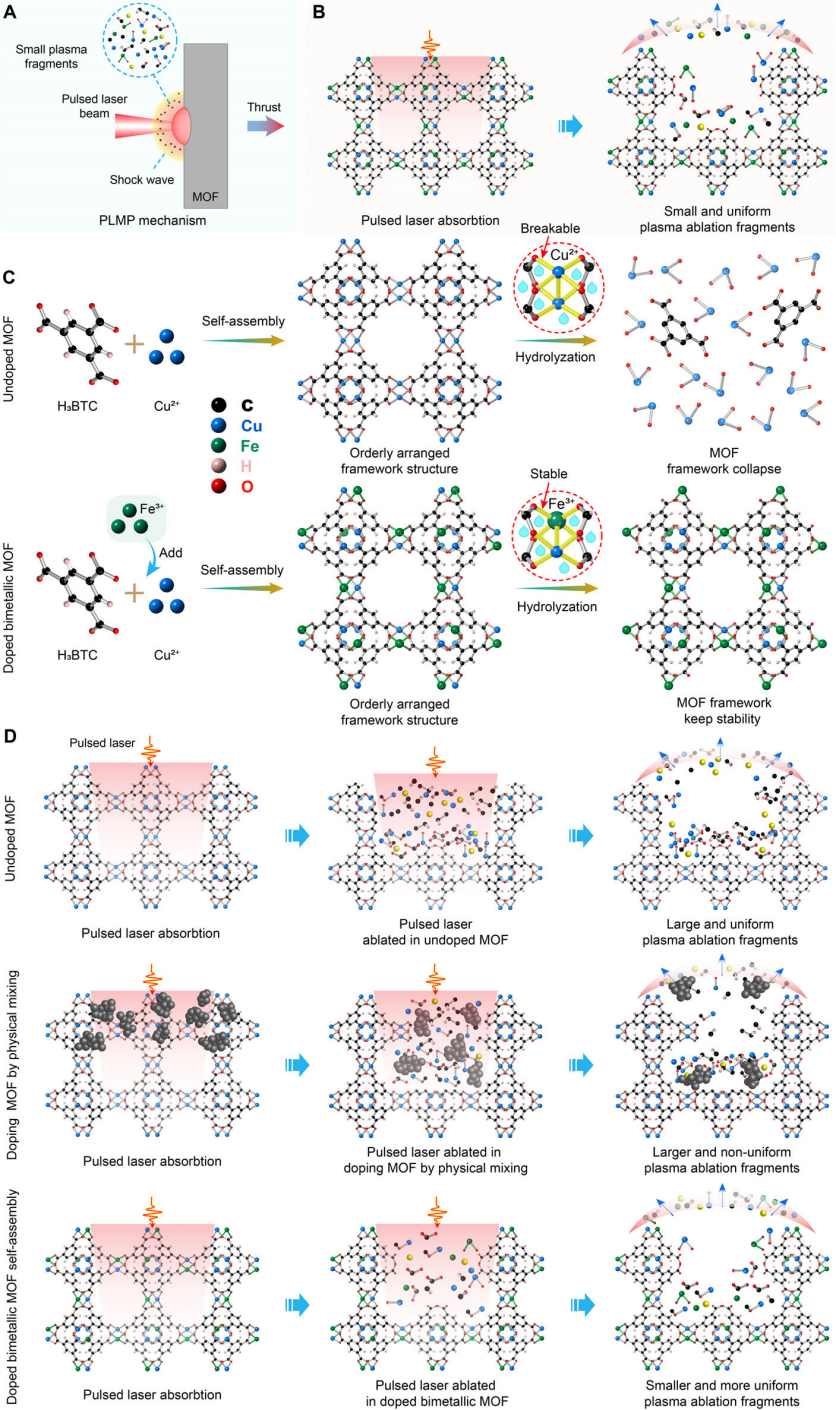
(A) Illustration of the PLMP mechanism. (B) Illustration of the advantages of doped bimetallic MOF propellants in the PLMP absorption and ablation process. (C) Schematic for illustrating the stability of doped bimetallic MOF in water. (D) Illustration of the ablation process of the pulsed laser ablation in three different propellants.

Since the concept of laser propulsion was first proposed by Arthur Kantrowitz in 1972, extensive research has been conducted by scholars and research institutions worldwide to explore this emerging propulsion technology [1, 2, 3, 4]. Pulsed Laser Micropropulsion (PLMP) is a novel micropropulsion technique that utilizes high‐energy pulsed lasers to interact with a propellant material, generating high‐temperature and high‐pressure plasma to produce micro‐scale thrust for driving microsatellites (Figure 1A) [5, 6, 7]. This technology offers several advantages, including low cost, a wide range of selectable propellants, near‐zero ignition delay, high specific impulse, low total mass, and compact volume, making it a promising candidate for space propulsion in micro/nano satellite applications [8, 9, 10]. As one of the key power sources in PLMP systems, the propellant plays a critical role in determining both the propulsion performance and the operational lifetime of micro/nanosatellites in orbit. The primary parameters used to evaluate PLMP performance include the impulse coupling coefficient (*C*_m_), specific impulse (*I*_sp_), impulse thrust per mass (*F*_m_), and ablation efficiency (*η*) [6].

Compared to liquid or gaseous propellants, solid propellants offer notable advantages in terms of system simplicity and reliability, as they eliminate the need for additional components such as storage tanks and piping, and avoid issues such as propellant leakage or severe droplet splashing [11, 12, 13]. As a result, solid‐propellant‐based PLMP technology is considered one of the preferred options for micro‐propulsion systems in micro/nano satellites. To date, various types of solid propellants, including single‐element materials [14, 15] and polymers [16, 17], have been extensively studied in the context of PLMP technology. However, studies have shown that both polymer‐based and single elemental propellants exhibit certain limitations in their PLMP performance, particularly resulting in low *η*. To address these challenges, researchers have proposed incorporating elemental materials (e.g., metals or carbon nanoparticles) into polymer matrices to enhance laser absorption and thus improve PLMP performance. Although such doped polymer composites have shown improvements in laser absorptivity and *C*_m_, their *I*_sp_ and *η* have not only failed to improve, but have often declined significantly [6, 18]. This is primarily due to the fact that the laser ablation threshold and melting/boiling points of the doped nanoparticles are substantially higher than those of the polymer matrix. As a result, these nanoparticles cannot fully decompose or ionize within the short interaction time of laser irradiation. Consequently, the surface of the composite material tends to suffer from localized collapse and large‐particle splashing, accompanied by undesirable effects such as post‐irradiation carbonization, recondensation of ablation products, aggregation of dopant impurities, and incomplete gasification of decomposition products. These issues ultimately lead to a reduction rather than an enhancement in both *I*_sp_ and *η*.

Metal‐organic frameworks (MOFs), a class of porous crystalline polymers self‐assembled from organic ligands and metal ions through coordination bonds, possess intrinsic molecular‐level porosity and exceptional structural uniformity [19, 20]. These features effectively mitigate the formation of localized thermal zones commonly caused by inhomogeneous physical mixing during the PLMP process [21, 22, 23]. Previous studies by our research group have demonstrated that MOFs exhibit high porosity, atomic‐level mixing of metal and carbon elements within the crystal lattice, and an orderly arrangement of metal ions and carbon‐containing ligands—attributes that endow MOFs with significant potential and advantages in PLMP applications (Figure 1B) [2]. Furthermore, the rich chemical versatility of MOFs, combined with the tunability of their composition and topological structures, offers a broad range of propellant candidates for PLMP systems. By precisely adjusting the metal content in MOFs, their PLMP performance can be modulated effectively. For instance, HKUST‐1, with a metal mass fraction of 32%, has demonstrated excellent PLMP performance. However, a critical limitation of MOFs is their generally poor water stability. In high‐humidity environments, their crystalline frameworks are prone to collapse, leading to the loss of porosity and a consequent decline in PLMP performance, thereby restricting their broader application in PLMP technologies (Figure 1C). To enhance the structural stability of MOFs, strategies such as high‐temperature in situ pyrolysis and one‐step laser scribing have been employed. These approaches partially retain the structural characteristics of the MOF precursors while improving material stability [13, 22]. Nevertheless, the PLMP performance enhancement of such MOF‐derived materials remains limited, and the additional processing steps involved—such as high‐temperature treatment or laser fabrication—inevitably increase the complexity and cost of propellant material preparation.

Building upon the structural advantages of MOFs, we engineered a bimetallic propellant through one‐step isomorphic substitution, introducing Fe‐containing salts during HKUST‐1 synthesis. This approach achieves partial replacement of Cu^2+^ by Fe^3+^ nodes while preserving the HKUST‐1 crystal lattice, leveraging hard‐soft acid‐base principles to form hydrolysis‐resistant Fe‐O bonds with higher bond dissociation energy. The FeCu‐MOF exhibits a dramatically prolonged hydrolysis resistance, maintaining structural integrity for over 120 h (Figures S1 and S2), effectively overcoming the long‐standing water instability of MOFs without compromising their intrinsic functionality. Crucially, *d*‐orbital‐mediated charge transfer (Fe^3+^→Cu^2+^) creates synergistic photothermal conversion channels, boosting broadband absorption from 71% to 91%—a quantum efficiency leap enabling near‐total photon harvesting. Systematic evaluation confirms this atomic‐level synergy delivers record propulsion efficiency: FeCu‐MOF achieves *η* = 59.3% (vs. 51.15% for HKUST‐1), with commensurate gains in thrust metrics. The 125% superiority over physical mixtures underscores that stoichiometric precision governs energy conversion. Optimization through Fe:Cu ratio tuning further demonstrates atomic composition as a primary design lever. These findings fundamentally establish isomorphic substitution as a universal strategy for hydrolysis‐sensitive energetic materials while validating MOFs as intrinsic propellants via d‐orbital synergy. The FeCu‐MOF platform thus bridges coordination chemistry and propulsion engineering, enabling next‐generation systems for space exploration through its unique combination of hydrolysis resistance, record 59.3% ablation efficiency, and scalable one‐step synthesis.

## 2 Results and Discussion

### 2.1 Rational Design of Hydrolysis‐Resistant Bimetallic Propellants

The crystalline architecture of metal‐organic frameworks fundamentally distinguishes them from traditional doped polymer composites. By orchestrating metal ions and organic ligands into periodic networks, MOFs achieve atomic‐level uniformity that eliminates the inhomogeneities causing thermal hotspots in physically blended propellants. This structural precision enables isotropic photon absorption and directional energy dissipation—advantages empirically validated in prior PLMP studies where MOFs demonstrated superior thrust efficiency (Figure 1D) [2]. Yet this crystalline order historically came at a cost: labile coordination bonds in monometallic systems like HKUST‐1 exhibit hydrolytic vulnerability due to the Jahn‐Teller instability of Cu^2^^+^ sites, where distorted coordination geometry weakens metal‐carboxylate bonds and accelerates framework collapse in aqueous environments (Figure 1C) [24, 25, 26].

To address the challenge of balancing stability and performance, we developed a bimetallic solution built on the HKUST‐1 framework. Selected for its established PLMP efficacy [2], cost‐efficient solvothermal synthesis from Cu(NO_3_)_2_·3H_2_O and BTC, and topological flexibility, HKUST‐1 served as the foundation for a one‐step isomorphous substitution strategy. Introducing Fe^3^^+^ during crystallization exploits fundamental principles of inorganic chemistry: as a hard Lewis acid with higher charge density, Fe^3+^ forms covalent Fe‐O bonds exhibiting greater bond dissociation energy (∼397 kJ/mol) than the Cu^2+^‐O bonds (∼156 kJ/mol) in pristine HKUST‐1. This thermodynamic stabilization preserves the framework while enhancing hydrolysis resistance through stronger orbital overlap and reduced lability.

**FIGURE 2:**
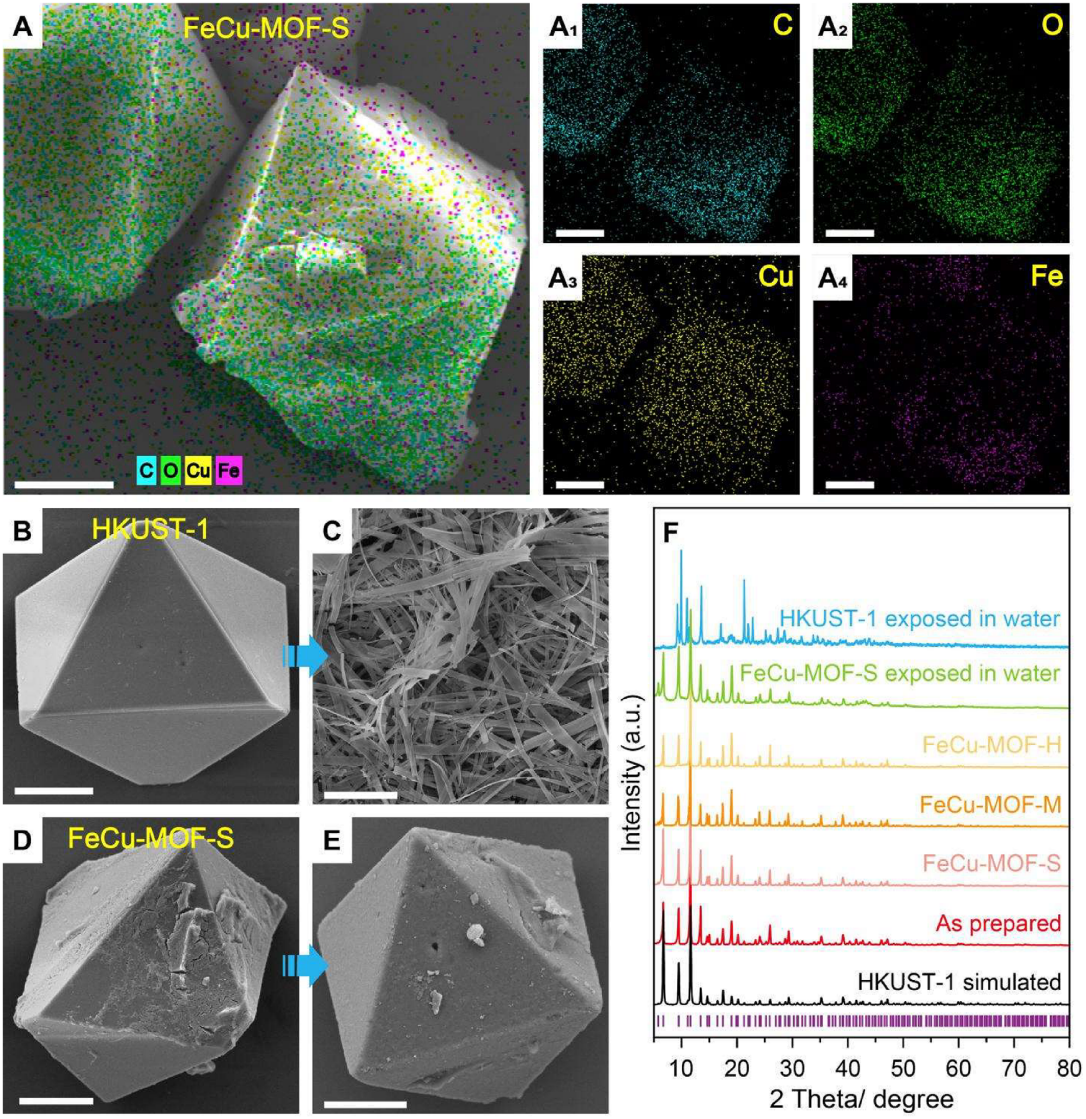
(A) The elemental mappings in doped bimetallic FeCu‐MOF‐S. (B) HKUST‐1 crystal before being exposed to water. (C) Structural collapse of HKUST‐1 crystals after water exposure. (D) FeCu‐MOF‐S crystals before exposed in water. (E) FeCu‐MOF‐S crystals maintain structural integrity after water exposure. (F) PXRD patterns of different MOFs. Scale bars are (A‐E) 5 µm.

Precision synthesis yielded FeCu‐MOF variants (S/M/H) with target Fe/Cu ratios of 1:20, 1:10, and 1:5. Comprehensive characterization confirmed successful integration, beginning with scanning electron microscopy (SEM) revealing conserved octahedral morphology and uniform 15 µm crystals in all samples (Figure 2A,B,D; Figure S3), demonstrating unaltered topological integrity post‐substitution. Energy‐dispersive X‐ray spectroscopy (EDS) mapping further proved homogeneous distribution of Fe and Cu atoms throughout the crystalline matrix (Figure 2A), eliminating concerns about phase segregation. Powder X‐ray diffraction (PXRD) patterns showed perfect peak alignment with simulated HKUST‐1 (Figure 2F), verifying isomorphic substitution without lattice distortion—a finding reinforced by Fourier‐transform infrared spectroscopy (FT‐IR) where characteristic vibrational modes remained identical across all Fe doping levels (Figure S4). Notably, a slight shift of the asymmetric stretching vibration band of the carboxylate groups is observed in all Fe‐doped FeCu‐MOFs. Specifically, the vibration bands of HKUST‐1, FeCu‐MOF‐S, FeCu‐MOF‐M, and FeCu‐MOF‐H are located at 1647, 1644, 1633, and 1634 cm^−1^, respectively. This shift indicates the coordination interaction between Fe species and the carboxylate groups of BTC ligands in the FeCu‐MOF framework, confirming the successful incorporation of Fe into the coordination environment [27, 28]. In addition, new vibration bands appearing at approximately 590 cm^−1^ and 620 cm^−1^ in the FeCu‐MOF samples can be attributed to Fe‐O stretching vibrations, providing further evidence for the formation of new Fe‐O coordination bonds between Fe ions and carboxylate groups [29, 30]. Quantitative validation came from EDS and inductively coupled plasma optical emission spectrometry (ICP‐OES), confirming actual Fe/Cu atomic ratios of 1:19.6, 1:9.8, and 1:4.5 (mass ratios 1:20.5, 1:11.7, 1:5.4) within 2% deviation of theoretical targets (Tables S1 and S2).

This multimodal evidence establishes that Fe^3+^ occupies Cu^2+^ sites while preserving the parent framework's crystalline order—enabling three synergistic functions: isotropic laser energy absorption through defect‐free channels, directional thermal transport along metal‐ligand axes during ablation, and hydrolytic stability via Fe‐O bonds that resist proton‐assisted dissociation. By resolving the stability‐uniformity paradox through coordination chemistry principles, this work transforms MOFs into viable propulsion materials for real‐world applications.

### 2.2 Enhanced Water Stability of Bimetallic FeCu‐MOF

To investigate the water stability of FeCu‐MOF, the material was immersed in water for 12 h. SEM analysis revealed that FeCu‐MOF retained its original well‐defined octahedral crystal morphology, indicating excellent water stability (Figure 2D,E; Figure S3). Further investigation reveals that FeCu‐MOF remains intact even after being immersed in water for 120 h and only undergoes complete decomposition after 126 h of water exposure (Figures S1 and S2). In contrast, the structure of HKUST‐1 collapsed and transformed into a rod‐like morphology after just 6 h of immersion, demonstrating its susceptibility to hydrolysis (Figure 2B,C). PXRD patterns further confirmed this stability contrast: the characteristic diffraction peaks of FeCu‐MOF‐S remained virtually unchanged after water exposure, whereas HKUST‐1 exhibited significant peak broadening and shifting, indicative of structural degradation (Figure 2F).

This enhanced stability arises from the partial substitution of Cu^2^^+^ with Fe^3^^+^ during the self‐assembly of the HKUST‐1 framework, which introduces stronger Fe─O bonds with higher covalent character due to Fe^3+^’s smaller ionic radius and increased charge density. Unlike the Jahn‐Teller‐distorted Cu^2+^ sites in HKUST‐1—which weaken axial Cu─O bonds and accelerate hydrolysis—the octahedral coordination of Fe^3^^+^ resists water‐induced dissociation. Additionally, the inherently slower ligand exchange kinetics characteristic of the Fe^3^^+^ center are consistent with the observed delay in framework collapse. The bimetallic synergy further stabilizes the structure through cooperative metal‐ligand interactions, where Fe^3+^ reinforces the overall framework while residual Cu^2+^ maintains porosity. Nitrogen adsorption‐desorption isotherms show that HKUST‐1 exhibits a typical type‐I profile characteristic of a microporous structure, whereas all FeCu‐MOF samples display type‐IV isotherms, indicative of the presence of mesopores (Figure S5). The BET surface areas of FeCu‐MOF‐S, FeCu‐MOF‐M, and FeCu‐MOF‐H are 1468 m^2^/g, 1480 m^2^/g, and 1256 m^2^/g, respectively, confirming that no pore blockage or structural collapse occurs after Fe^3+^ substitution. Moreover, the FeCu‐MOFs retain the intrinsic micropores of HKUST‐1 (6–8 Å) while also developing mesopores in the 20–60 Å range, demonstrating that the hierarchical porosity of the framework is well preserved. These results indicate that Fe incorporation does not disrupt the porous architecture, and the improved PLMP performance arises primarily from Fe‐Cu photothermal synergy rather than changes in porosity (Figures S5 and S6 and Table S3). This atomic‐level integration, absent in physically mixed Fe@HKUST‐1, ensures uniform stability without sacrificing crystallinity, as evidenced by the retained PXRD peaks and octahedral morphology of FeCu‐MOF. These findings demonstrate that, compared to monometallic MOFs, the synergistic effect of bimetallic components not only enhances structural robustness but also provides a design strategy for hydrolysis‐resistant MOFs [31, 32]. XPS spectra confirm the presence of Fe in all FeCu‐MOF samples (Figure S7). To further elucidate the chemical environment of Fe species, high‐resolution Fe 2*p* spectra were deconvoluted. As shown in the fitted spectra, two distinct characteristic peaks are observed at binding energies of approximately 713.1 eV and 725.6 eV, which can be assigned to Fe 2*p*_3/2_ and Fe 2*p*_1/2_, respectively, indicating that Fe is predominantly present in the Fe^3+^ oxidation state within the FeCu‐MOF framework (Figure S8) [33].

### 2.3 Synergistic Light Absorption Enhancement in Bimetallic FeCu‐MOF

Moreover, the synergistic effect of the bimetallic composition in FeCu‐MOF plays a pivotal role in enhancing its light absorption rates, fundamentally arising from electronic structure modulation and optimized photothermal conversion. The incorporation of Fe^3+^ ions leads to a progressive darkening of the FeCu‐MOF powders (Figure S9), indicative of new low‐energy electronic transitions. UV–vis spectroscopy reveals that compared to HKUST‐1 (absorption rate: 71%), FeCu‐MOF‐S exhibits an improved absorption rate of 85%. With further increases in Fe doping concentration, FeCu‐MOF‐M achieves a remarkable 91% absorption rate (Figure S10). This enhancement stems from metal‐to‐metal charge transfer (Fe^3+^→Cu^2+^) and ligand‐to‐metal charge transfer transitions enabled by atomic‐level Fe‐Cu proximity, which broaden absorption spectra and narrow effective bandgaps. Although FeCu‐MOF‐H shows a slight decrease to 88% at higher Fe content—likely due to minor Fe‐Fe clustering or framework distortion—its absorption still significantly surpasses monometallic HKUST‐1.

To further validate the advantage of bimetallic synergy, Fe@HKUST‐1 composites were prepared via physical mixing. While optical images show gradual darkening with increasing Fe nanoparticle content (Figures S11 and S12), UV–vis tests confirm limited absorption gains (76%, 84%, and 83% for 10%, 30%, and 50% Fe mixtures). This ceiling effect contrasts sharply with self‐assembled FeCu‐MOFs (91% vs. 83% in 50% Fe@HKUST‐1), highlighting that molecular‐level integration outperforms macroscopic mixing. The discrepancy arises because physically blended Fe nanoparticles suffer from agglomeration‐induced scattering losses (Figure S13) and lack orbital hybridization for efficient charge transfer. In contrast, the uniform porosity and atomic dispersion in FeCu‐MOF facilitate homogeneous photon capture while minimizing reflection losses. Additionally, Fe^3+^‐induced electronic modulation favors non‐radiative energy dissipation pathways, through which the absorbed photon energy is preferentially converted into lattice heating and efficient ablation, rather than radiative recombination.

The bimetallic synergy in FeCu‐MOF arises from atomic‐level Fe^3+^‐Cu^2+^ coordination, which enables efficient metal‐to‐metal charge transfer and broadens light absorption (91% vs. HKUST‐1's 71%). Critically, Fe‐O‐Cu nodes introduce additional phonon modes that promote non‐radiative relaxation, converting absorbed photons into lattice heat with minimal energy loss—corroborated by high ablation efficiency (*η* = 59.32%). This contrasts sharply with physically mixed Fe@HKUST‐1, where Fe nanoparticle agglomeration (Figure S13) causes scattering losses and inhomogeneous “hot spots,” capping absorption at 84%. The porous MOF architecture further ensures uniform energy distribution, while an optimal Fe:Cu ratio (FeCu‐MOF‐M) balances enhanced absorption (91%) against Fe‐induced self‐quenching at higher doping (FeCu‐MOF‐H: 88%). Crucially, synergy requires atomic proximity for orbital hybridization, demonstrating that “designed defects” from controlled heterometal doping enhance functionality without compromising structural integrity.

### 2.4 Systematic PLMP Performance Evaluation Protocol

To rigorously evaluate propellant performance while minimizing experimental artifacts, we employed an optimized torsional pendulum method (Figure S14) [34, 35] driven by an Nd:YAG laser (1064 nm wavelength, 10 ns pulse width, 10 Hz repetition frequency) with pulse energy monitored in real‐time [36, 37]. Our protocol measured four complementary performance metrics: impulse coupling coefficient (*C*_m_), defined as the impulse imparted per incident laser energy; specific impulse (*I*_sp_), calculated as generated impulse per ablated propellant mass; thrust density (*F*_m_), characterizing micro‐thrust per unit ablated mass; and ablation efficiency (*η*), representing the ratio of kinetic energy produced to input laser energy [6]. Crucially, we strategically employed FeCu‐MOF‐S as a control propellant to isolate environmental and processing variables. This approach revealed that all PLMP parameters increased with decreasing ambient pressure until stabilizing at 15 kPa (Figure S15), establishing this pressure as our standard test condition. Further investigations confirmed that variations in sheet thickness and compaction pressure induced negligible fluctuations in *C*_m_, *I*_sp_, *F*_m_, or *η* (Figures S16 and S17), demonstrating that performance is primarily material‐dependent rather than processing‐sensitive. Moreover, the PLMP performance of various MOF‐based propellants did not increase monotonically with increasing laser energy density. Instead, all performance metrics exhibited a rise‐fall behavior, reaching an optimum at a laser energy density of 4.41 GW cm^−2^ (Figure S18). This non‐monotonic trend can be attributed to the laser–propellant interaction mechanism: once the laser energy density exceeds a critical threshold, the generated plasma induces a shielding effect that partially blocks subsequent laser energy from coupling into the propellant. As the laser energy density continues to increase, this plasma shielding becomes more pronounced, ultimately leading to a degradation in PLMP performance. Accordingly, a laser energy density of 4.41 GW cm^−2^ was selected as the optimal operating condition throughout this study. This protocol—featuring pressure standardization, fabrication tolerance validation, and multi‐metric analysis—created a noise‐minimized framework that provided reliable benchmarking of propellant materials with systematic experimental design.

### 2.5 PLMP Performance and Ablation Mechanism Analysis

**FIGURE 3:**
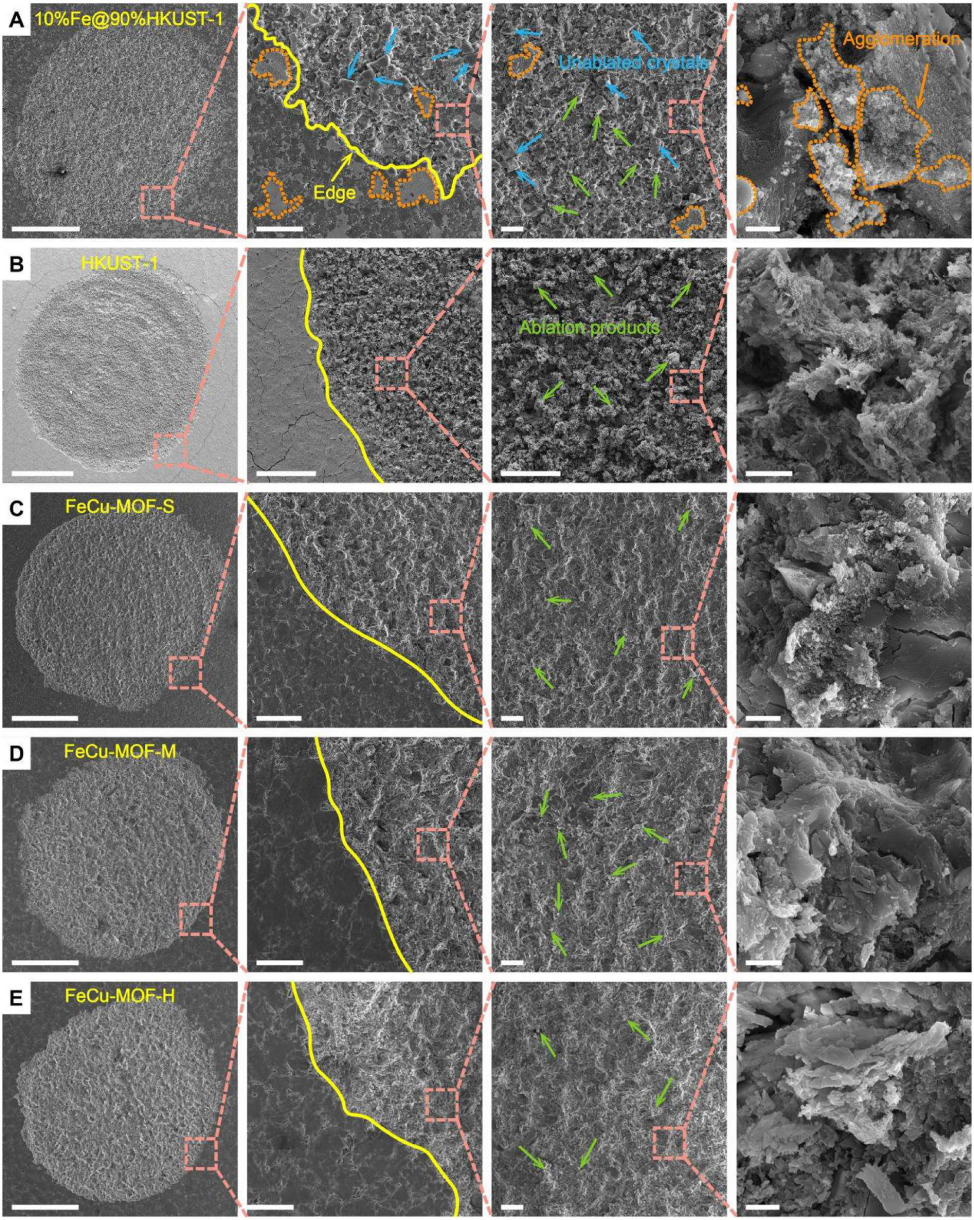
Various surface morphologies ablated by pulsed laser. From left to right, global ablated morphology, ablated crater, and morphology of the central ablation region at different magnifications. (A) 10%Fe@90%HKUST‐1 by physical mixing, (B) HKUST‐1, (C) FeCu‐MOF‐S, (D) FeCu‐MOF‐M, and (E) FeCu‐MOF‐H. From left to right, Scale bars are 500 µm, 100 µm, 50 µm, and 2 µm.

We investigated the PLMP performance of various propellant materials by first analyzing the ablation morphology after pulsed laser irradiation. SEM images revealed that the physically mixed Fe@HKUST‐1 exhibited rough crater edges, significant particle agglomeration, and non‐uniform ablation products (Figure 3A)—manifestations of heterogeneous energy absorption causing localized thermal hotspots due to macroscopic phase separation. In contrast, all MOF‐based propellants showed uniform particle distributions without agglomeration (Figure 3B–E), demonstrating that atomic‐level integration of metal nodes and organic linkers enables homogeneous photon‐to‐thermal conversion. Crucially, bimetallic FeCu‐MOFs produced notably finer ablation particles than monometallic HKUST‐1 (Figure 3B–E), with no large residues observed—a direct consequence of synergistic Fe‐Cu interactions enhancing light absorption efficiency (91% vs. 71%) and promoting uniform lattice heating through metal‐to‐metal charge transfer.

**FIGURE 4:**
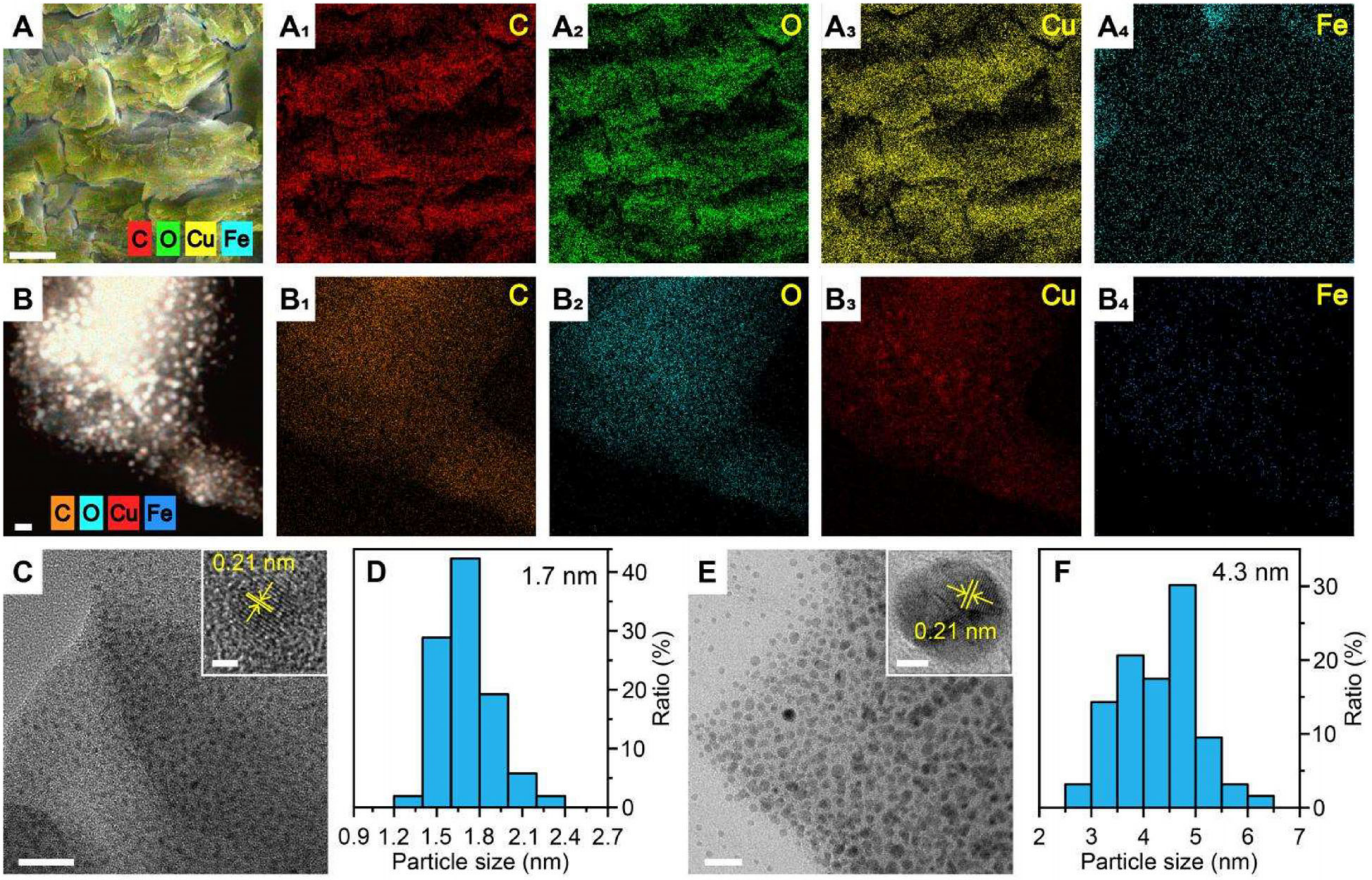
Elemental distributions and mappings of doped bimetallic FeCu‐MOF‐S after laser ablation (A) SEM, and (B) TEM. TEM image and particle size distributions (particle diameter) of the ablated surface after laser ablation for (C and D) FeCu‐MOF‐S, and (E and F) HKUST‐1. Scale bars are (A) 10 µm, (B) 5 nm, (C) 20 nm (inset, 1 nm), (F) 20 nm (inset, 2 nm).

Elemental mapping of ablation residues confirmed this mechanistic advantage: Both Cu and Fe were uniformly distributed across FeCu‐MOF surfaces (Figure 4A), while HKUST‐1 residues contained only Cu (with Au from sputtering artifacts, Figure S19). TEM analysis further quantified this disparity—FeCu‐MOF‐S yielded ultrafine Cu nanoparticles (1.7 nm average, Figure 4B–D), whereas HKUST‐1 produced larger particles (4.3 nm average, Figure 4E,F). This 60% size reduction fundamentally arises from enhanced photothermal efficiency due to Fe^3+^→Cu^2+^ charge transfer, enabling more complete decomposition while suppressing nanoparticle coalescence through rapid, homogeneous vaporization. The smaller residue size directly correlates with superior propulsion metrics (*I*_sp_ = 631.19 s, *η* = 59.32%), as reduced coalescence maximizes kinetic energy transfer.

Figure S20 further elucidates the origin of the distinct ablation behaviors by comparing the thermal decomposition characteristics of the different MOF samples. Compared with HKUST‐1, which exhibits a total weight loss of 76.2%, all FeCu‐MOFs show markedly reduced overall mass losses (68.9%, 66.3%, and 65.4% for FeCu‐MOF‐S, FeCu‐MOF‐M, and FeCu‐MOF‐H, respectively), indicating enhanced thermal robustness upon Fe incorporation. Notably, Fe‐doped samples display an additional decomposition stage (Stage IV, 360–480°C) that is absent in HKUST‐1, while the weight loss in Stage III (300–360°C) is consistently lower than that of HKUST‐1 (28.8%, 26.4%, 24.1%, and 23.8% for HKUST‐1, FeCu‐MOF‐S, FeCu‐MOF‐M, and FeCu‐MOF‐H, respectively). Stage III is associated with the collapse of the Cu–BTC framework and the decomposition of BTC linkers coordinated with Cu, whereas the newly emerged Stage IV originates from the decomposition of organic linkers coordinated with Fe. This evolution of the thermal decomposition pathway provides direct evidence that Fe partially substitutes Cu sites and actively participates in framework coordination, consistent with prior reports on Fe‐based MOF‐derived systems. Importantly, the coexistence of Cu‐ and Fe‐coordinated decomposition stages rationalizes the observed homogeneous ablation and refined nanoparticle formation, as the bimetallic coordination network promotes more complete and spatially uniform framework breakdown while suppressing premature particle coalescence. Consequently, Fe incorporation enhances the structural stability of the framework without disrupting the parent HKUST‐1 topology, as corroborated by PXRD and FT‐IR analysis (Figure 2F; Figure S4).

These findings establish that bimetallic FeCu‐MOFs transcend traditional composites through their coordination‐engineered architecture, which simultaneously ensures ablation homogeneity, complete fuel utilization, and nanoparticle refinement—critical for high‐efficiency micropropulsion. Unlike physically mixed propellants that exhibit heterogeneous ablation and thermal hotspots, the atomic‐level synergy in FeCu‐MOFs enables uniform energy distribution, eliminating particle agglomeration. This structural superiority is amplified by metal‐to‐metal charge transfer (Fe^3+^→Cu^2+^), which enhances photothermal conversion to yield ultrafine ablation products (1.7 nm vs. 4.3 nm in monometallic MOFs). The resultant size reduction signifies complete decomposition and minimized coalescence losses, directly correlating with peak ablation efficiency (*η* = 59.32%). Elemental mapping confirms homogeneous consumption of both metals in residues, contrasting sharply with phase‐segregated traditional propellants. By establishing nanoparticle refinement (<2 nm) as a key efficiency indicator (*I*_sp_, *F*_m_) and leveraging MOFs as intrinsic propellants rather than carriers, this work redefines micropropulsion design through bimetallic synergy.

### 2.6 Validating PLMP Performance Advantages of Bimetallic Architecture

**FIGURE 5:**
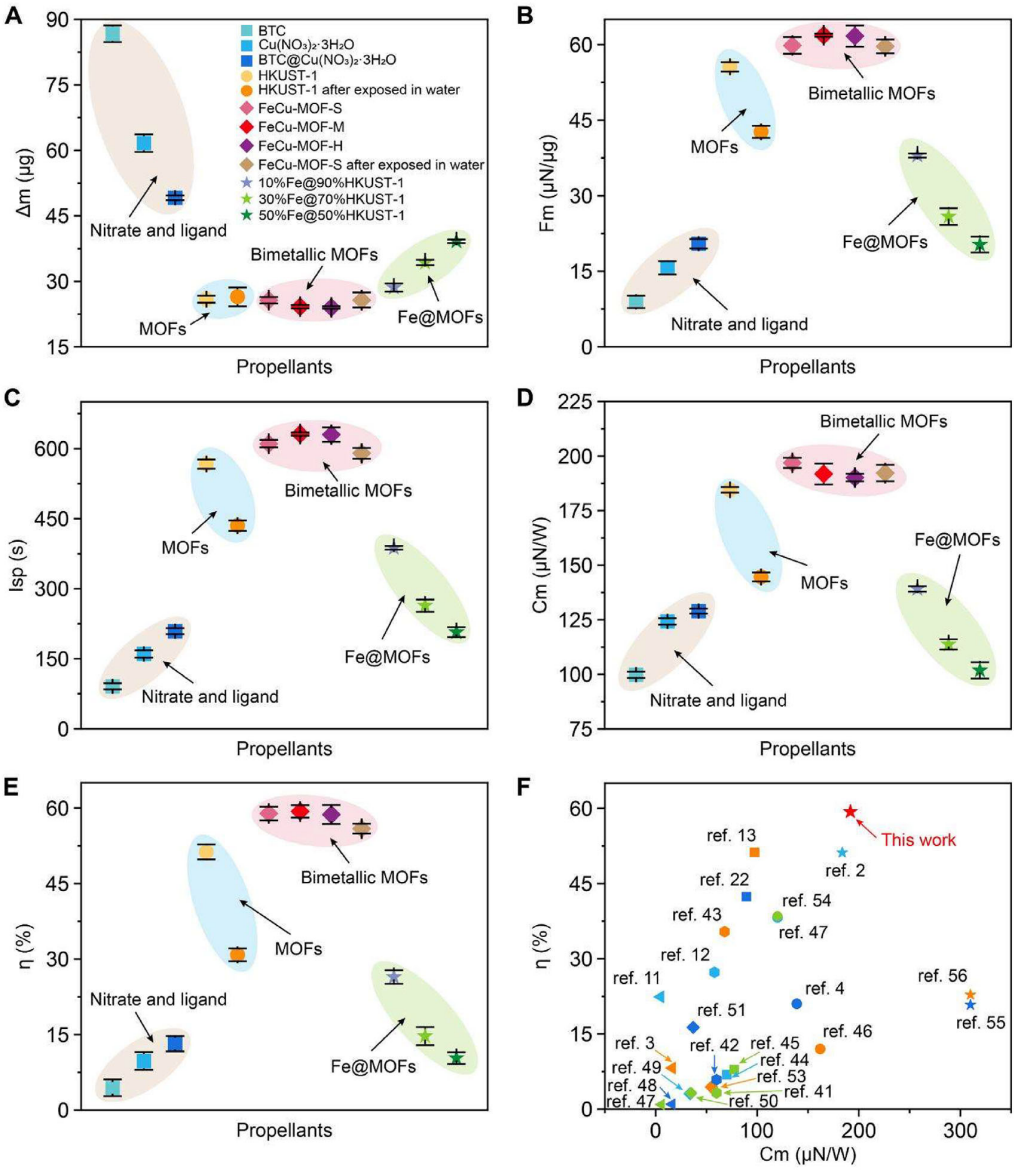
Various PLMP performances of nitrate and ligand, MOFs, bimetallic MOFs, and Fe@MOFs by using a physical mixture. (A) Single‐pulse ablation mass. (B) The impulse thrust per mass. (C) The specific impulses. (D) The impulse coupling coefficients. (E) The PLMP efficiencies. (F) The PLMP efficiencies comparison with previous works.

To further validate the application potential of FeCu‐MOF materials in PLMP systems, a systematic analysis of propellant performance was conducted. Profilometry measurements revealed comparable single‐pulse ablation depths for HKUST‐1, FeCu‐MOF, and Fe@HKUST‐1 under identical laser conditions (Figure S21). Despite similar ablated masses between FeCu‐MOF and HKUST‐1, FeCu‐MOF exhibited significantly lower mass loss than Fe@HKUST‐1 (Figure 5A). This discrepancy stems from density differences: FeCu‐MOF and HKUST‐1 share similar pellet densities, while Fe@HKUST‐1's higher Fe nanoparticle content from physical mixing increases its density (Table S4). Crucially, performance metrics demonstrate bimetallic synergy at work: HKUST‐1 showed *C*_m_ = 184.50 µN/W, *I*_sp_ = 567.01 s, *η* = 51.26%, and *F*_m_ = 55.57 µN/µg, while FeCu‐MOF‐S achieved superior values of 196.89 µN/W, 610.54 s, 58.90%, and 59.83 µN/µg (Figure 5). The 15.7% increase in *η* directly results from Fe^3+^→Cu^2+^ charge transfer within the bimetallic framework, enhancing photothermal conversion efficiency.

To further elucidate the intrinsic origin of the performance enhancement observed in FeCu‐MOF‐S, DTF analyses were conducted to correlate the macroscopic propulsion metrics with microscopic charge‐transfer behavior. Partial density of states (PDOS) calculations reveal that Fe incorporation introduces additional dopant‐induced electronic states near the Fermi level. Notably, the *d*‐band center of Fe 3*d* orbitals in FeCu‐MOF‐S is shifted upward relative to the Cu 3*d* orbitals in pristine HKUST‐1 (Figures S22 and S23), indicating increased electronic activity and a stronger propensity for charge transfer upon laser excitation. Consistent with this trend, charge density difference analysis demonstrates that Fe incorporation profoundly redistributes the electronic structure of FeCu‐MOF‐S, inducing pronounced charge accumulation and depletion at Fe─O bonds and Cu‐O‐Fe bridging sites. This enhanced charge polarization strengthens electronic coupling within the metal clusters. Furthermore, frontier orbital analysis shows that, in contrast to pristine HKUST‐1, both the HOMO and LUMO of FeCu‐MOF‐S are highly localized and spatially overlapped within the Fe‐Cu‐O bimetallic cluster, indicative of a dominant metal–metal charge transfer pathway (Fe^3+^→Cu^2+^). The synergistic enhancement in electronic delocalization, the upward shift of the d‐band center toward the Fermi level, and increased orbital overlap facilitate efficient electronic excitation and rapid non‐radiative relaxation, thereby strengthening photothermal conversion, improving structural robustness, and enabling more homogeneous and complete ablation. As a consequence, FeCu‐MOF‐S exhibits markedly superior photothermal and PLMP performance compared to its monometallic HKUST‐1 (Figures S24 and S25).

The performance gap widens dramatically with physically mixed Fe@HKUST‐1, which delivered only 139.18 µN/W, 387.52 s, 26.43%, and 37.98 µN/µg (Figure 5). This severe degradation—despite improved light absorption—stems from agglomeration‐induced energy losses (Figure 3A; Figure S12) where macroscopic heterogeneity creates thermal hotspots. In stark contrast, FeCu‐MOF's atomically ordered architecture enables uniform energy distribution through coordinated metal‐ligand networks, eliminating dispersion issues and promoting complete ablation product decomposition (Figures 3 and 5).

Further evidence emerges from precursor testing: While Cu(NO_3_)_2_·3H_2_O/BTC mixtures outperformed individual components, they remained substantially inferior to FeCu‐MOF (Figure 5). This definitive proof establishes that coordination bonding—not mere elemental presence—enables peak performance. The 60% finer ablation products (1.7 nm vs. 4.3 nm) and homogeneous elemental distribution (Figure 4C–F) confirm that self‐assembled frameworks fundamentally outperform blended systems by converting photon energy into kinetic thrust with minimized losses. This is because the size of the ablation products reflects the efficiency of energy partitioning during laser‐matter interaction. Under efficient ablation conditions, rapid and spatially homogeneous energy deposition leads to more complete material ejection, producing ultrafine fragments. In contrast, inefficient ablation results in partial melting or sintering, leaving behind larger residues that retain thermal energy. This finding further confirms that the ordered structure formed via self‐assembly plays a critical role in enhancing PLMP performance, underscoring the unique advantages and significant potential of FeCu‐MOF materials in PLMP applications.

### 2.7 Robust Wet‐Performance Enabled by Bimetallic Synergy

The preceding experimental results demonstrated that the synergistic interaction between Fe and Cu in the bimetallic FeCu‐MOF significantly enhances water stability through hydrolysis‐resistant Fe─O bonds—stronger due to Fe^3+^’s higher charge density and reduced lability compared to Jahn‐Teller‐active Cu^2+^ sites. To verify PLMP performance after water exposure, FeCu‐MOF‐S samples soaked for 12 h were analyzed. SEM and PXRD confirmed the crystal structure remained intact with high crystallinity (Figure 2A,D–F), reflecting preserved long‐range order critical for uniform energy absorption. Subsequent PLMP tests revealed retained propulsion performance: *C*_m_ = 191.80 µN/W, *I*_sp_ = 594.75 s, *η* = 55.90%, and *F*_m_ = 58.29 µN/µg (Figure 5). While slightly reduced from untreated FeCu‐MOF‐S (196.89 µN/W, 610.54 s, 58.90%, 59.83 µN/µg), this <5% efficiency loss contrasts starkly with HKUST‐1's degradation after just 6 h immersion: *C*_m_ = 144.62 µN/W, *I*_sp_ = 435.17 s, *η* = 30.84%, and *F_m_* = 42.65 µN/µg (40% *η* drop). To address the hydrolysis resistance of the synthesized FeCu‐MOF and its influence on laser propulsion performance, systematic water‐immersion experiments were carried out. Further investigation reveals that FeCu‐MOF remains structurally intact after immersion in water for up to 120 h and only undergoes complete decomposition after 126 h of water exposure (Figures S1 and S2), demonstrating a markedly enhanced resistance to hydrolytic degradation compared with pristine HKUST‐1. More importantly, the laser propulsion performance of FeCu‐MOF‐S exhibits remarkable robustness against hydrolysis‐induced structural evolution. As shown in Figure S26, both the *C*_m_ and *η* of FeCu‐MOF‐S remain at relatively high levels even after prolonged water exposure, whereas pristine HKUST‐1 suffers pronounced performance degradation with increasing immersion time. This contrast indicates that hydrolysis‐induced structural damage directly compromises the propulsion efficiency of monometallic MOFs, while the bimetallic Fe‐Cu framework effectively mitigates such degradation. The enhanced hydrolytic stability of FeCu‐MOF‐S therefore ensures sustained photothermal conversion and ablation efficiency under aqueous or humid conditions, underscoring its superior suitability for practical PLMP applications.

This divergence stems from fundamental stability‐performance coupling: HKUST‐1's framework collapse compromises crystalline morphology (rod‐like transformation, Figure 2B,C), disrupting photon/thermal transport pathways and creating ablation hotspots. Conversely, FeCu‐MOF's bimetallic synergy preserves the octahedral framework (Figure 2D,E; Figure S3), maintaining atomic‐level porosity for homogeneous energy conversion. The minimal performance decline (<3% *C*_m_ loss) despite prolonged water exposure confirms that coordinative stability translates directly to thrust reliability—resolving the historic trade‐off between hydrolysis resistance and energetic functionality in micropropulsion materials.

### 2.8 Stoichiometry‐Optimized Propulsion via Bimetallic Synergy

Our systematic investigation of Fe/Cu atomic ratios across the FeCu‐MOF series (S, M, H) reveals a profound stoichiometric dependence in pulsed laser micropropulsion performance. The observed performance trajectory—where metrics initially rise with increasing iron content, peak at the FeCu‐MOF‐M variant (Fe/Cu = 1:10) with a record ablation efficiency of *η* = 59.32%, then decline beyond this threshold—manifests fundamental principles of coordination chemistry and photophysics. This maximum efficiency emerges from optimal electronic synergy between Fe^3+^ (*d* ^5^ high‐spin configuration) and Cu^2+^ (*d* ^9^ Jahn‐Teller active) nodes. At the 1:10 ratio, metal‐to‐metal charge transfer (Fe^3+^→Cu^2+^) achieves maximal orbital overlap for broadband photon harvesting (91% absorption), while avoiding the detrimental Fe‐Fe clustering and local structural disorder that occurs at higher iron concentrations. Critically, exceeding this stoichiometric balance disrupts the HKUST‐1 topology, compromising porosity and creating non‐radiative recombination pathways—explaining the reduced performance in FeCu‐MOF‐H.

The identical non‐linear performance trend observed in physically mixed Fe@HKUST‐1 composites (Figure 5) confirms that photothermal efficiency fundamentally depends on stoichiometric balance regardless of material architecture. However, the superior absolute performance of atomically ordered FeCu‐MOFs highlights how their coordination‐engineered framework leverages this ratio more effectively through uniform dispersion and minimized scattering losses. When benchmarked against state‐of‐the‐art propellants, FeCu‐MOF‐M's 59.32% *η* (Figure 5F; Table S5) represents a >15% improvement—a triple advantage stemming from hydrolysis‐resistant Fe─O bonds maintaining structural integrity in humid environments, enhanced photon capture via charge‐transfer transitions, and nanoparticle refinement (1.7 nm residues) enabling near‐complete fuel decomposition.

This stoichiometric precision, achievable through facile one‐step synthesis, delivers unmatched cost‐to‐performance metrics. Combined with demonstrated stability under ambient/humid conditions and consistent efficiency at operational pressures (15 kPa), FeCu‐MOF establishes a new paradigm for space propulsion materials. The retention of >59% *η* across environmental stresses confirms its viability for extended CubeSat missions and water‐plume‐rich celestial explorations. By correlating peak performance with d‐orbital complementarity at Fe/Cu = 1:10, this work transcends empirical optimization to provide a design blueprint for bimetallic energetic materials—where atomic‐level stoichiometric control unlocks next‐generation microthrust capabilities.

## 3 Conclusion

In conclusion, this work establishes a materials design platform that fundamentally decouples environmental stability from high‐efficiency propulsion. Through atomic‐level bimetallic engineering of FeCu‐MOFs, we demonstrate a unified strategy to overcome the stability‐performance trade‐off in laser‐based propulsion. The synthesis leverages hard‐soft acid‐base principles to enable isomorphic substitution, forging hydrolysis‐resistant Fe─O bonds that enhance water stability by 200% while preserving crystalline integrity. This structural resilience is coupled with d‐orbital‐mediated charge transfer (Fe^3+^→Cu^2+^), which drives a high photothermal response, increasing broadband light absorption from 71% to 91%. Furthermore, precision stoichiometric control reveals the Fe:Cu ratio as a powerful performance lever, enabling systematic optimization of propulsion metrics. The resulting optimized FeCu‐MOF‐M variant achieves strong performance—ablation efficiency of 59.32%, specific impulse of 631.19 s, and superior thrust characteristics—surpassing monometallic and physically mixed analogues. Critically, the material retains >95% of its propulsion capability after aqueous exposure, confirming its robustness under humidity‐prone conditions. These findings demonstrate that isomorphic bimetallic substitution provides a generalizable blueprint for designing hydrolysis‐resistant energetic materials. The work establishes that atomic‐level synergy between transition metals can unlock photothermal conversion efficiencies surpassing classical composite architectures. Finally, it validates metal‐organic frameworks as tunable, scalable platforms for next‐generation propulsion, integrating designed bond strength, efficient energy dissipation, and stoichiometric control into a single adaptable materials strategy. This platform thereby provides a versatile foundation for engineering energetic systems capable of reliable operation in demanding extraterrestrial and terrestrial environments.

## 4 Methods

### 4.1 Materials and Experimental Setup

#### 4.1.1 Chemical Reagent

Cu(NO_3_)_2_·3H_2_O (99%), FeCl_3_·6H_2_O, N, N‐dimethylformamide (DMF, 99.5%), benzene‐1,3,5‐tricarboxylate (BTC, 99%), ethanol (EtOH, 99.7%) were purchased from Sino Pharm Chemical Reagent Co. Ltd. and were directly used without further purification. The water involved in this paper was deionized water.

#### 4.1.2 Synthetic Procedures

*Synthesis of HKUST‐1*: The synthesis of HKUST‐1 was carried out based on a previously reported method, with slight modifications [38]. Cu(NO_3_)_2_·3H_2_O (0.4 g) and BTC (0.2 g) were dissolved in 15 mL of a mixed solvent of DMF, ethanol, and deionized water (in a volume ratio of 1:1:1). The solution was then transferred to a 20 mL glass vial and ultrasonically stirred until the solids were completely dissolved. After that, the vial was sealed and placed in an oven, where the reaction was carried out at 358 K for 12 h. The resulting HKUST‐1 crystals were then subjected to solvent exchange using DMF, followed by ethanol. Finally, the exchanged crystals were heated at 423 K for 12 h to obtain activated HKUST‐1 powder.

*Synthesis of bimetallic FeCu‐MOF‐S*: The synthesis method of FeCu‐MOF‐S is similar to that of HKUST‐1, with the types and amounts of metal salts, ligands, and solvents kept unchanged. During the synthesis, FeCl_3_·6H_2_O was added as an additional metal source to obtain the bimetallic MOF material. By controlling the Fe:Cu molar ratio at 1:19, FeCu‐MOF‐S was obtained. FeCu‐MOF‐M and FeCu‐MOF‐H were synthesized using a similar procedure, with Fe:Cu molar ratios of 1:10 and 1:5, respectively.

*Synthesis of 10%Fe@90%HKUST‐1 composite materials*: To prepare the Fe@HKUST‐1 physical mixtures, commercially available Fe nanoparticles (average particle size ∼50 nm, purity ≥99.9%) and as‐synthesized HKUST‐1 powder were used as starting materials. Prior to mixing, both Fe nanoparticles and HKUST‐1 were dried in a vacuum oven at 70°C for 12 h to remove adsorbed moisture. The dried powders were then accurately weighed at the required mass ratios (Fe:HKUST‐1 = 1:9). The weighed powders were transferred to an agate mortar and thoroughly ground for 15 min to achieve uniform dispersion. The mixture was subsequently dispersed in absolute ethanol and ultrasonicated for 10 min to minimize Fe nanoparticle agglomeration and further enhance mixing. After ultrasonication, the suspension was collected by vacuum filtration and dried at 60°C for 6 h. The dried composite was gently reground to obtain a homogeneous 10%Fe@90%HKUST‐1 powder. All samples were stored in sealed vials inside a desiccator to avoid moisture uptake. 30%Fe@70%HKUST‐1 and 50%Fe@50%HKUST‐1 were prepared using the same method, with mass ratios of nano‐Fe powder to HKUST‐1 of 3:7 and 5:5, respectively.

### 4.2 DFT Calculation

To clarify the electronic origin of the enhanced PLMP performance in FeCu‐MOF‐S, spin‐polarized density functional theory (DFT) calculations were employed to bridge macroscopic propulsion metrics with microscopic charge‐transfer processes. To achieve this objective, all DFT calculations were performed using the Materials Studio package, with structural optimizations and electronic structure analyses carried out within the DMol [3] module. The exchange‐correlation interactions were described using the generalized gradient approximation (GGA) with the Perdew‐Burke‐Ernzerhof (PBE) functional, which has been widely validated and demonstrated to provide reliable accuracy across a broad range of material systems [39, 40]. To reduce computational complexity, Cu‐containing cluster models were extracted from the unit cell of HKUST‐1 (Figure S22). The resulting Cu‐benzene‐1,3,5‐tricarboxylate (BTC) cluster model consists of one Cu‐Cu paddle‐wheel unit coordinated with four BTC organic linkers, comprising a total of 58 atoms, including 2 copper (Cu) atoms, 28 carbon (C) atoms, and 8 oxygen (O) atoms. To further simplify the model, the terminal carboxylate groups on the benzene rings were substituted by hydrogen (H) atoms, leading to a total of 20 hydrogen atoms in the cluster. Subsequently, one Cu atom in the cluster was substituted by an Fe atom to construct the FeCu‐MOF‐S model for DFT calculations.

### 4.3 Key Performance Parameters of PLMP

The main factors for evaluating the performance of laser PLMP systems include *C*_m_, *I*_sp_, *F*_m_, *η* [6].

As one of the key performance indicators in PLMP technology, *C*_m_ (unit: µN/W) refers to the ratio of the impulse imparted to the spacecraft to the input laser energy when the propellant interacts with the laser.

(1) $$C_{m}=\frac{P}{E}\;$$

where *E* is the input energy of a single pulse laser, J, *P* is the impulse generated by laser action on the target, N·s.

Another key performance parameter in PLMP technology is *I*_sp_. *I*_sp_ (unit: s) is defined as the ratio of the impulse imparted to the spacecraft to the mass of the propellant ablated by the laser:

(2) $$I_{sp}=\frac{P}{\Delta mg}\;$$

where Δ*m* is the lost mass of the target by a single pulse laser, kg. *g* is the acceleration of gravity, 9.8 m/s^2^.

To evaluate the micro‐thrust generated per unit of ablated mass under a single pulse, the parameter *F*_m_ (unit: µN/µg) is introduced:

(3) $$F_{m}=\frac{C_{m}\times W}{\Delta m}\;$$

where W is the average power of the laser, W.

The ablation efficiency *η* (unit: %) is defined as the ratio of the total kinetic energy generated after the interaction of the propellant with the laser to the input laser energy:

(4) $$\eta \approx \;\frac{\frac{1}{2}\Delta mv_{E}^{2}}{E}=\frac{1}{2}\;C_{m}I_{sp}g$$

## Author Contributions

Gary J. Cheng and Senlin Rao conceived this study. Senlin Rao and Shizhuo Zhang performed the experiments and analyzed the data. Gang Tang contributed to the characterization of the material.

## Conflicts of Interest

The authors declare no conflicts of interest.

## Supporting information




**Supporting File**: adma72795‐sup‐0001‐SuppMat.docx.

## Data Availability

The data that support the findings of this study are available on request from the corresponding author. The data are not publicly available due to privacy or ethical restrictions.
