# Supplementary material for: An Atomic‐Level Bimetallic MOF Platform Overcoming the Stability‐Performance Tradeoff for Laser Propulsion
Source: Adv Mater. 2026 Mar 13;38(20):e72795. doi: 10.1002/adma.72795 (PMC13054119; doi:10.1002/adma.72795)
Supplement: Supplementary file 1 — Supporting File: adma72795‐sup‐0001‐SuppMat.docx. [file ADMA-38-e72795-s001.docx]

**Supporting Information**

**An Atomic-Level Bimetallic MOF Platform Overcoming the Stability-Performance Tradeoff for Laser Propulsion**

*Senlin Rao,^1^ Gang Tang,^1^ Shizhuo Zhang,^2^ and Gary J. Cheng^*,3^*

^1^ Jiangxi Provincial Key Laboratory of Precision Drive and Equipment, Jiangxi University of Water Resources and Electric Power, Nanchang 330099, PR China

^2^ The Institute of Technological Sciences, Wuhan University, Wuhan 430072, PR China

^3^ School of Industrial Engineering, Purdue University, West Lafayette, IN 47906, USA

**Characterization Methods**

Scanning electron microscopy (SEM) images were acquired using a Thermo Fisher Scientific Teneo Volumescope, equipped with a field emission gun and a Trinity detector. The crystal structures were analyzed by Powder X-ray Diffraction (PXRD), conducted on a diffractometer operating in Bragg-Brentano geometry with Cu Kα radiation (*λ* = 1.54178 Å) at 40 kV and 40 mA. PXRD data were collected over a 2θ range of 5 to 80° at a scanning rate of 10 °/min using Panalytical Data Collector software. Transmission electron microscopy (TEM) was performed on a JEOL 2100F instrument operating at an accelerating voltage of 200 kV. UV-vis-NIR spectra were recorded using a PerkinElmer Lambda 750S spectrophotometer, equipped with an integrating sphere to enhance measurement accuracy. The concentrations of Fe and Cu in FeCu-MOFs were measured by ICP-OES. The spectral range spanned from 200 to 2,500 nm. Fourier-transform infrared spectroscopy (FT-IR) was employed to analyze the vibrational functional groups of all synthesized MOF materials, within a wavelength range of 500 to 4,000 cm^-1^. The ablation depth and surface morphology were characterized using a 3D optical surface profilometer. XPS was performed with a photoelectron spectrometer on an K-Alpha (Thermo Scientific). Thermogravimetric analysis (TGA) was carried out on a DTG-60 thermogravimetric analyzer (Shimadzu) under an N_2_ atmosphere with a flow rate of 20 mL min^-1^, over a temperature range from 30 to 800 °C.

**Laser processing:**

To evaluate and analyze the PLMP performance of different propellant materials, a torsional pendulum device is used for testing, a method that has been widely adopted ^1-6^. A schematic diagram and the working principle of the torsional pendulum system are shown in Figure 5a. In the experiment, an Nd:YAG laser with a wavelength of 1064 nm is used as the laser source. The laser has a pulse width of 10 ns and a frequency of 10 Hz. The maximum energy of a single laser pulse is approximately 0.9 J, and the pulse energy is measured by an energy meter. The initial laser spot diameter is 9.5 mm. Since the unfocused laser spot is relatively large, a focusing lens (focal length *f* = 200 mm, diameter *Φ* = 25.4 mm) is used to concentrate the laser energy, thereby achieving a higher laser energy density. The energy variation between laser pulses is less than 3%. The laser energy fluence used in the experiment was 4.41 GW cm^-2^. All propellant materials are compressed into circular discs using a YP-15T small hydraulic press under a pressing pressure of 22 MPa. Before pressing, the MOFs powder is activated in an oven, and after pressing, the discs are again activated in the oven. The diameter of each disc is 8 mm, and the thickness is approximately 0.2 mm.

The experiments are conducted under vacuum conditions, with the vacuum chamber evacuated to 15 kPa using a mechanical pump. During the impulse measurement using the torsional pendulum method, when the pulsed laser energy is focused on the propellant material on one side of the pendulum, the material absorbs the laser energy and generates a localized high temperature, causing surface vaporization. The vapor, along with the plasma produced by the ionization of air around the laser focal point, generates an outward shock wave. The recoil from the shock wave drives the material ^7-9^, causing the pendulum arm to deflect at a certain angle in the horizontal direction. At this point, a guiding laser mounted on the pendulum arm reflects off a mirror at the center of the arm and moves across a measuring scale parallel to the arm. The displacement S of the reflected laser spot is recorded in real time by a camera. Using this displacement and a specific formula $P=\frac{\mathrm{GT}\mathrm{Sd}^{4}}{128ARL}$, the impulse *P* generated by the laser acting on the propellant material can be calculated.

In the impulse formula, *G* is the shear modulus of the torsion wire, Pa. According to GB/T23935-2009, the shear modulus of beryllium bronze wire is 4.21×10^10^ Pa. *d* represents the diameter of the torsion wire, m. *J* denotes the polar moment of inertia of the torsion wire’s cross-section, m⁴. *S* is the displacement of the guiding laser on the measuring scale, m. *A* is the distance between the scale and the torsion pendulum, which are parallel to each other, m. *T* is the oscillation period of the torsional pendulum, taken as the average over 20 full oscillation cycles, s.

The torsion wire used in the torsional pendulum system is a beryllium bronze wire, chosen for its good ductility, with a diameter *d* = 0.15 mm. The effective length *L* of the torsion wire is 20.0 mm. To ensure proper tension in the torsion wire, a weight is attached to the bottom of the wire and vertically immersed into molten paraffin. Once the paraffin solidifies, the wire gains a certain level of tension, effectively minimizing the influence of vertical forces on the measurement. To ensure the pendulum arm is both lightweight and strong, a hollow stainless steel square tube is used. Its length is 260 mm, so the effective arm length for impulse application is *R* = 130 mm. Using an electronic balance and a vernier caliper, the mass and thickness of the compressed propellant discs can be measured, allowing calculation of the density *ρ*_ₘ_ of each type of material. The densities of the various propellant discs are shown in Table S1. Since the mass ablated by a single laser pulse is extremely small, it is difficult to measure directly, even with a high-precision electronic balance, and the resulting measurement error is quite large ^10^. In this study, a white light interferometric 3D surface profiler is first used to measure the morphology and depth of the ablation craters from multiple single laser pulses. From this, the average ablation volume per pulse ($\bar{V}_{m}$) can be calculated, and thus the average ablated mass of the propellant per single laser pulse ($\bar{m}_{m}$) can be determined. Based on the impulse values measured by the torsional pendulum device and using equations (1)-(4), key LMP performance parameters of different propellant materials, such as *C*_m_, *I*_sp_, *F*_m_, *η,* can be obtained.


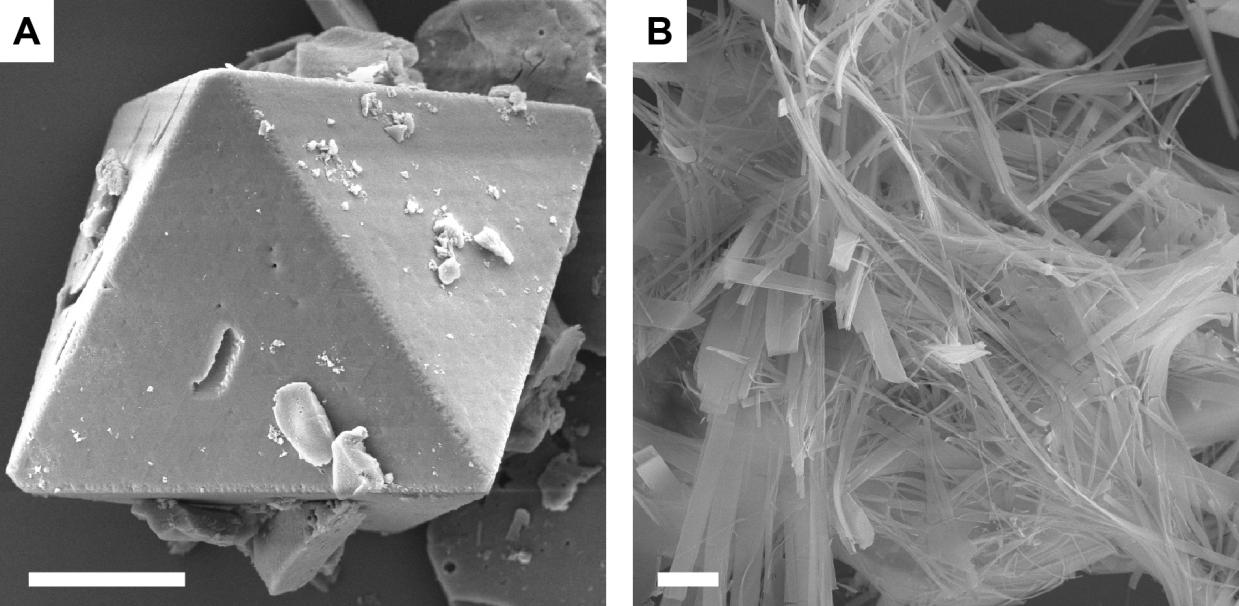


**Figure S1.** SEM images of FeCu-MOF-S after water exposure for (A) 120 h, and (B) 126 h.


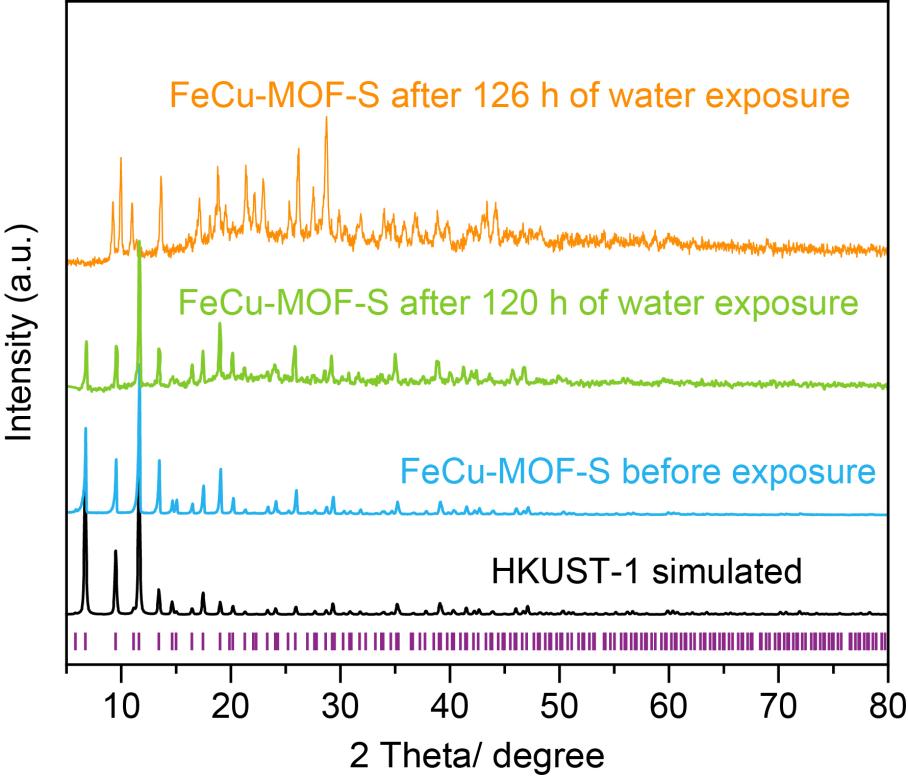


**Figure S2.** PXRD patterns of FeCu-MOF-S before and after water exposure.


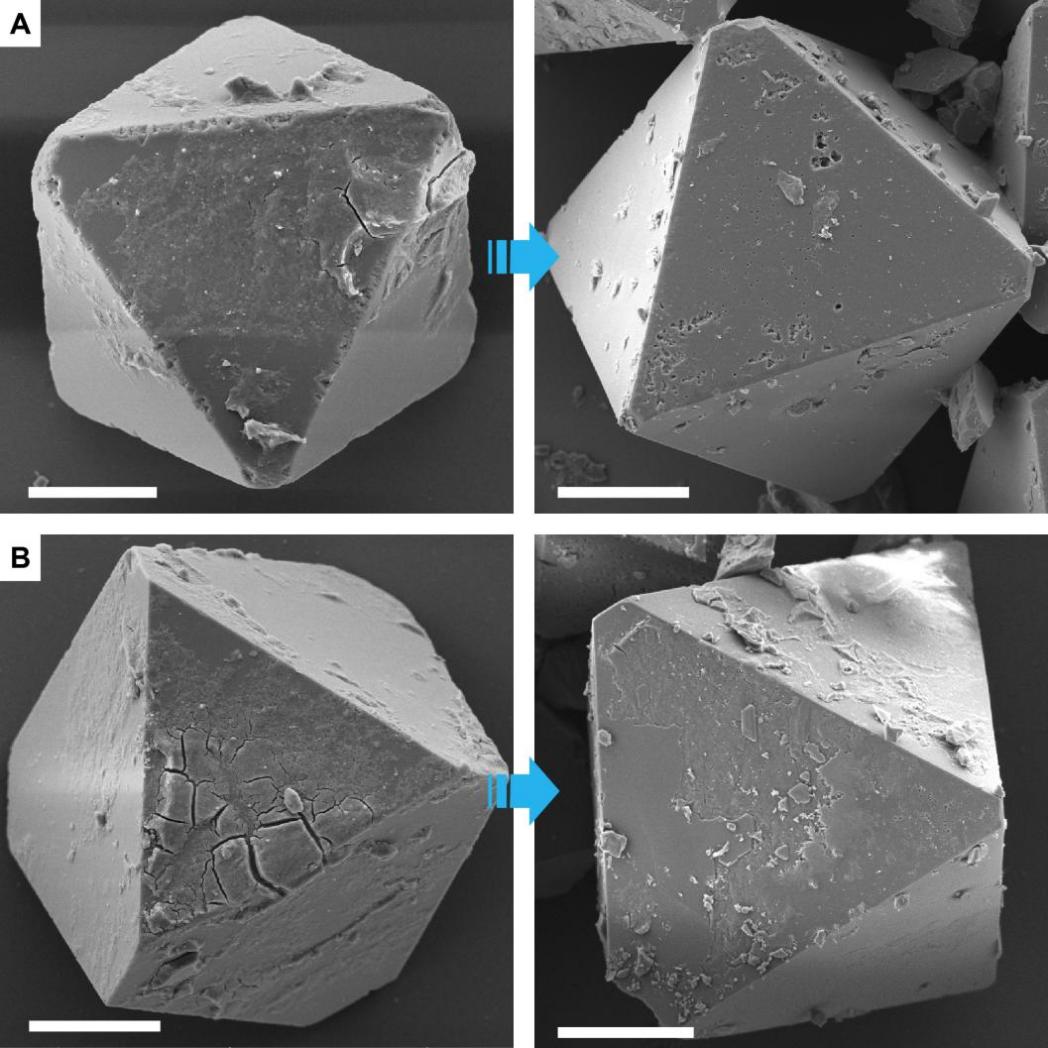


**Figure S3.** (A) FeCu-MOF-M crystals and (B) FeCu-MOF-H crystals maintain structural integrity after water exposure. Scale bars are 5 μm.


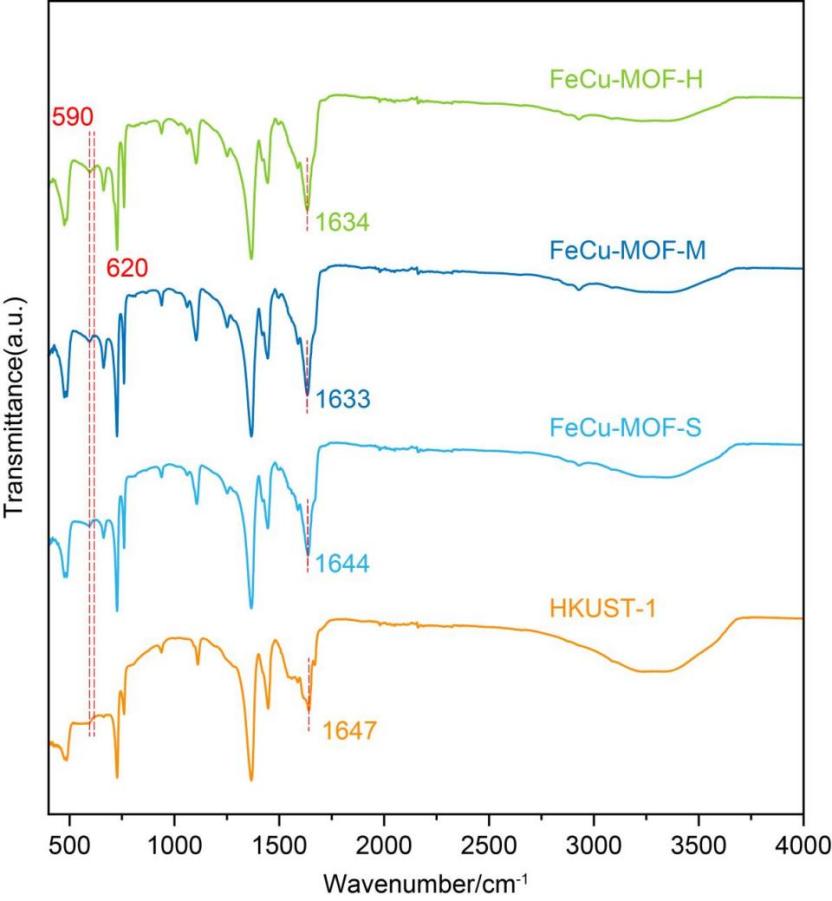


**Figure S4.** FT-IR spectra of HKUST-1, FeCu-MOF-S, FeCu-MOF-M and FeCu-MOF-H. In the Fe-doped FeCu-MOF samples, a slight shift of the asymmetric stretching vibration band of the carboxyl groups near 1647 cm^-1^ can be observed.


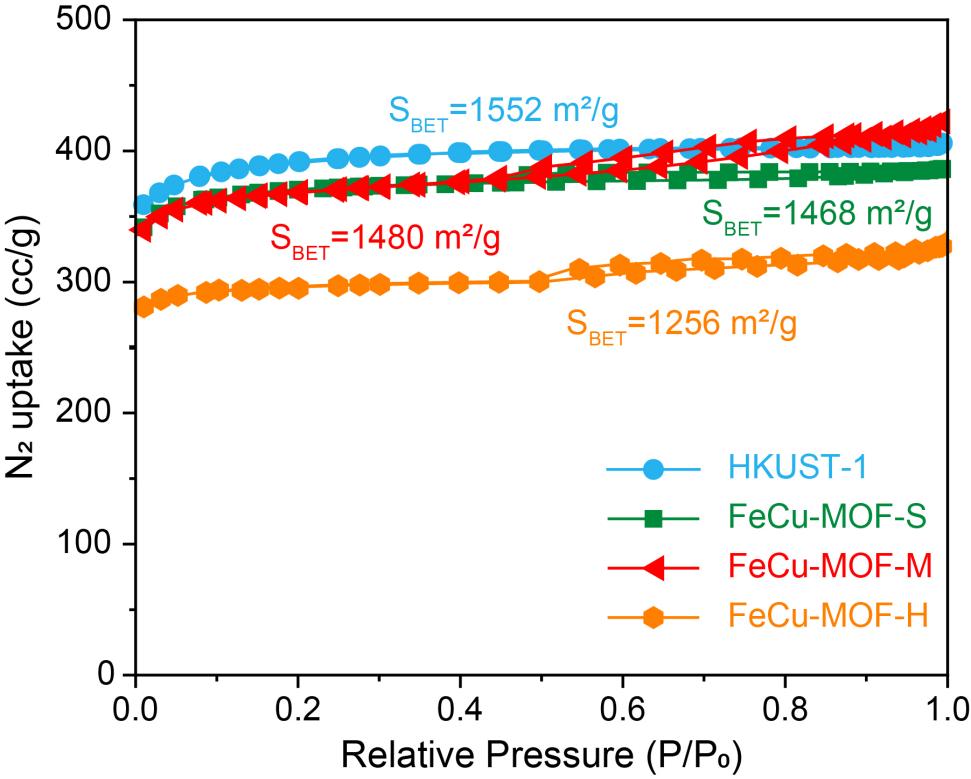


**Figure S5.**  N_2_ adsorption isotherms of the synthesized HKUST-1 and FeCu-MOF series samples.


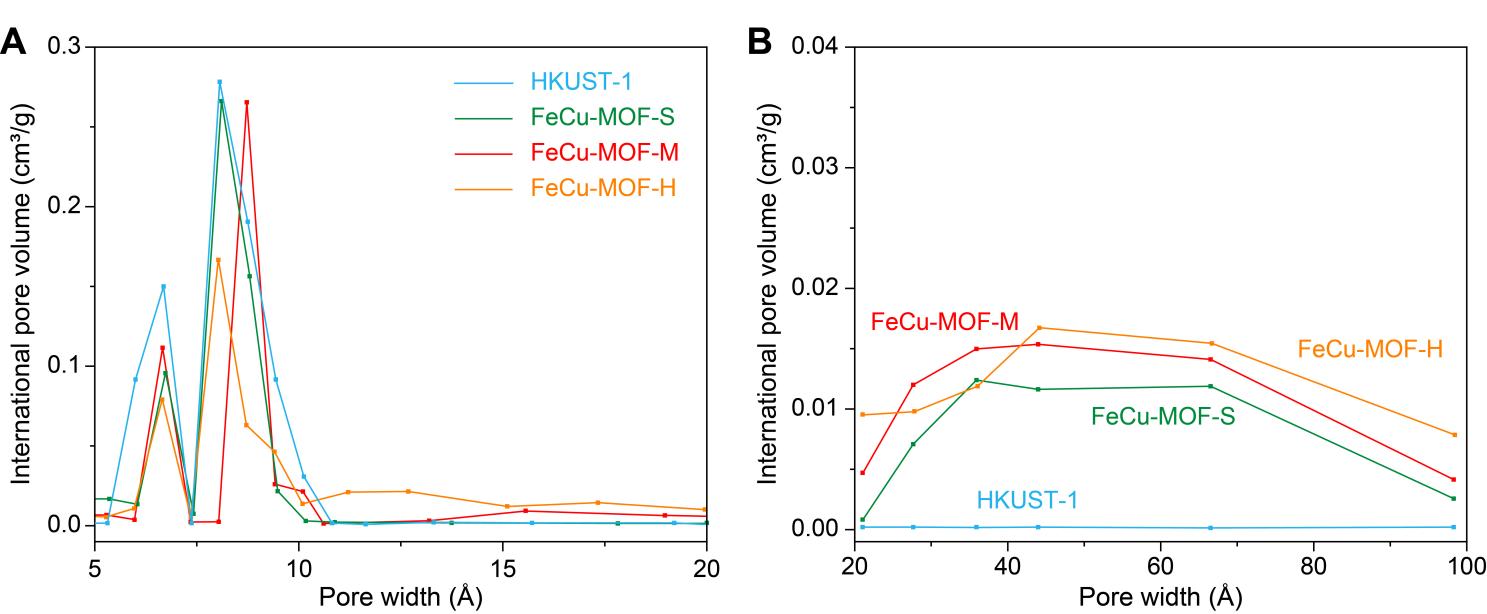


**Figure S6.** Pore size distributions of (A) micropore, (B) mesopore.


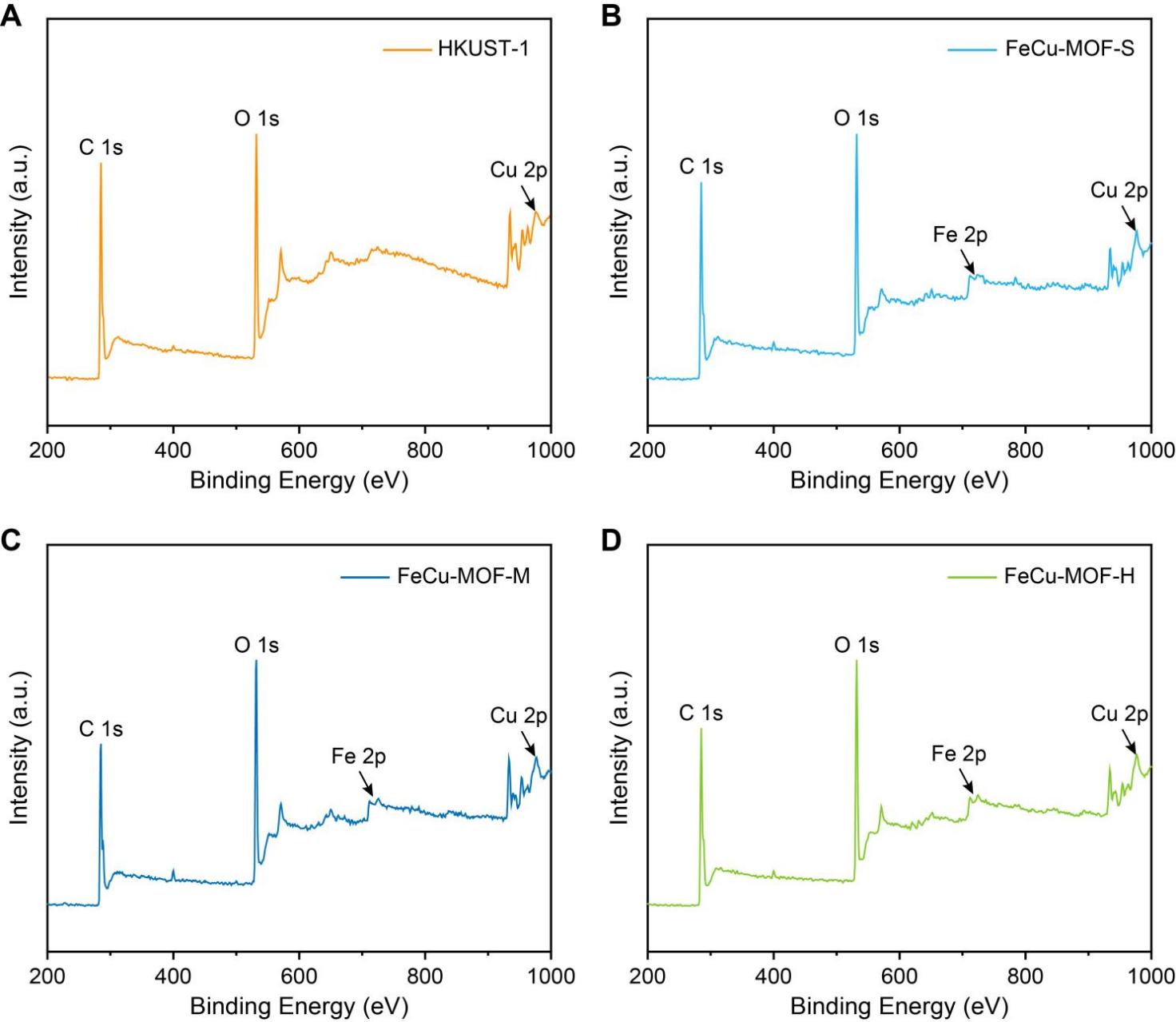


**Fugure S7.** Full survey XPS spectrum of (A) HKUST-1, (B) FeCu-MOF-S, (C) FeCu-MOF-M and (D) FeCu-MOF-H.


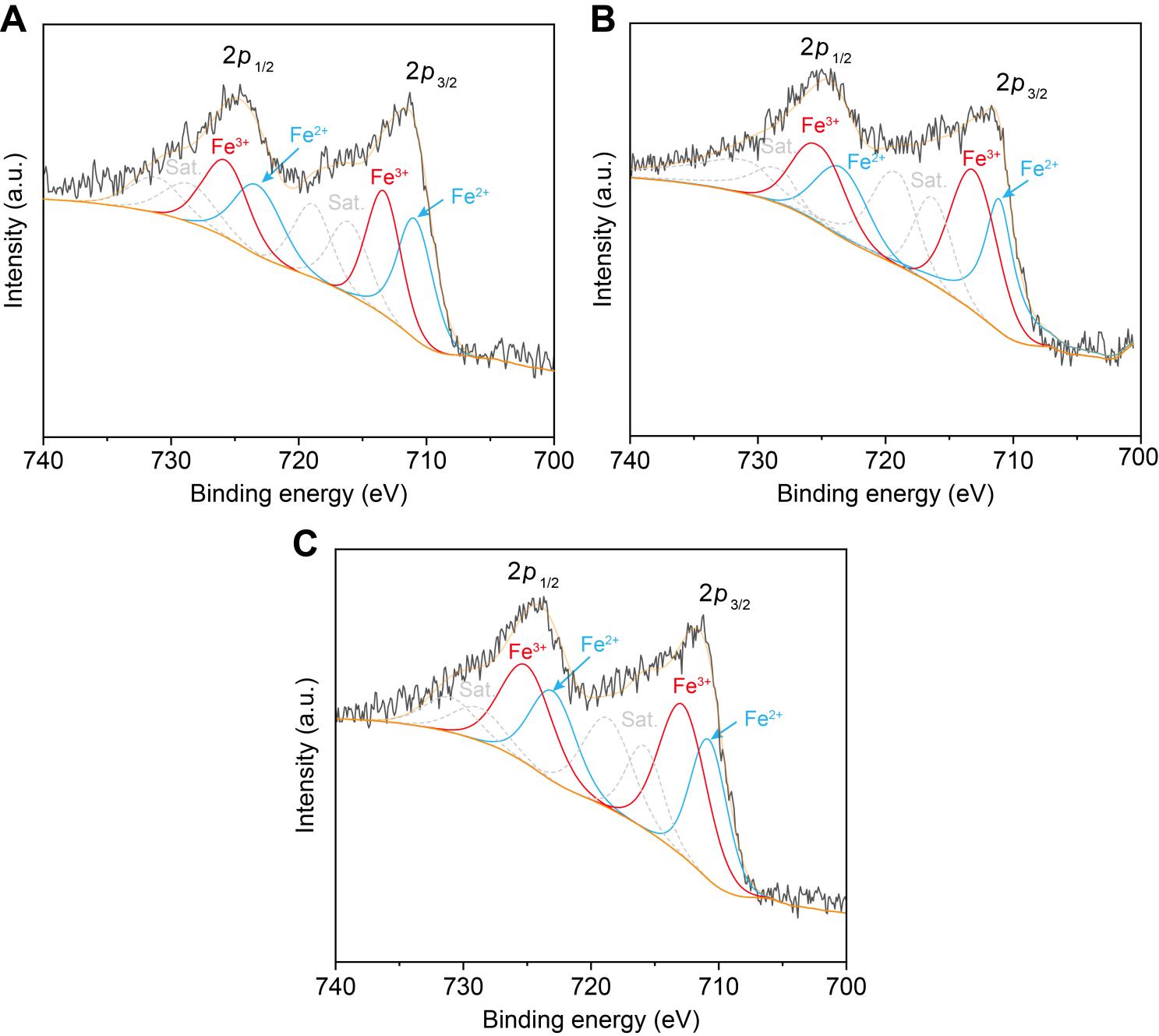


**Figure S8.** High-resolution Fe 2p XPS spectra of different FeCu-MOFs.


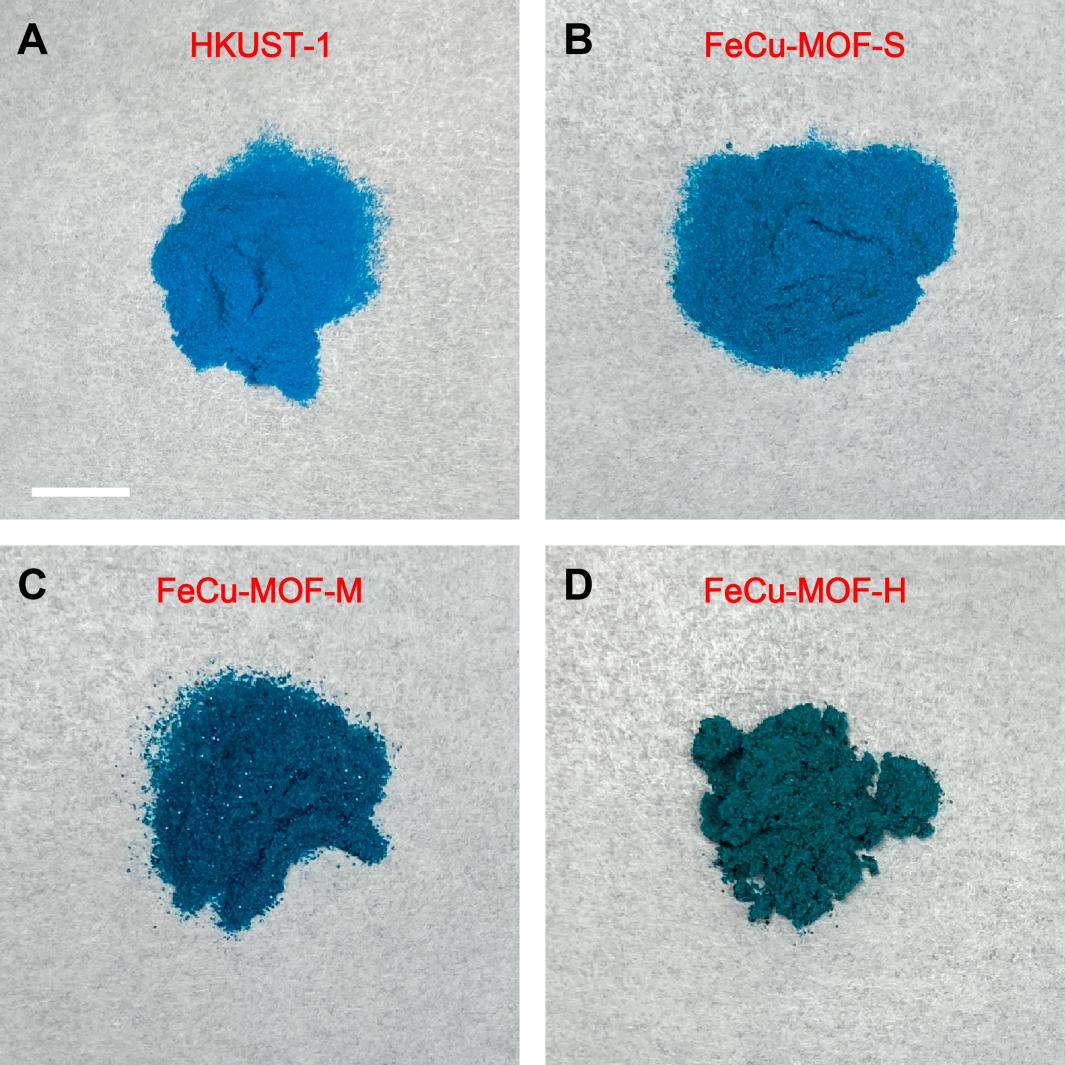


**Figure S9.** The optical image of (A)HKUST-1, (B) FeCu-MOF-S, (C) FeCu-MOF-M and (D) FeCu-MOF-H. Scale bars are (A-D) 2 cm.


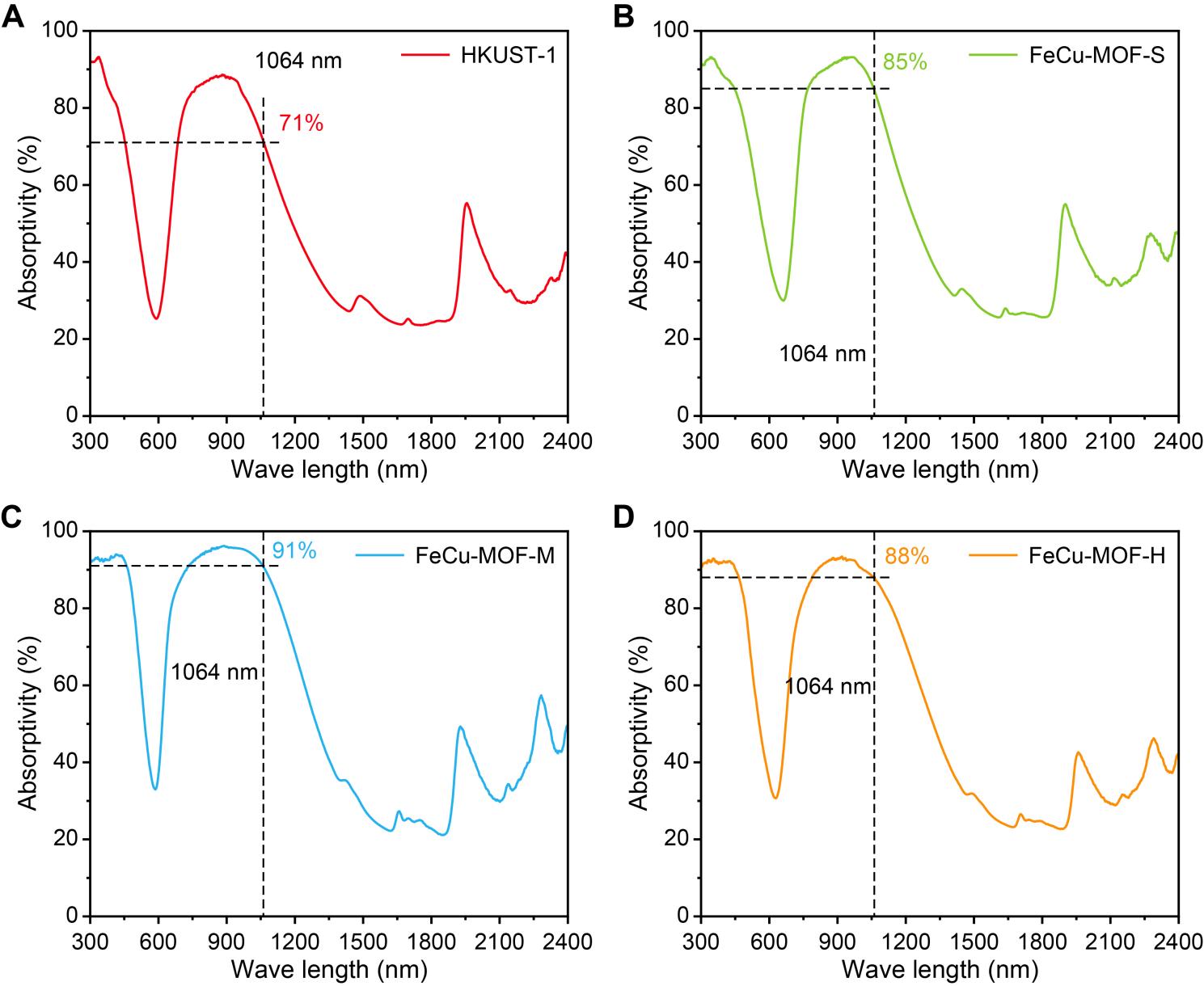


**Figure S10.** Experimental light absorption profiles of (A) HKUST-1, (B) FeCu-MOF-S, (C) FeCu-MOF-M and (D) FeCu-MOF-H.


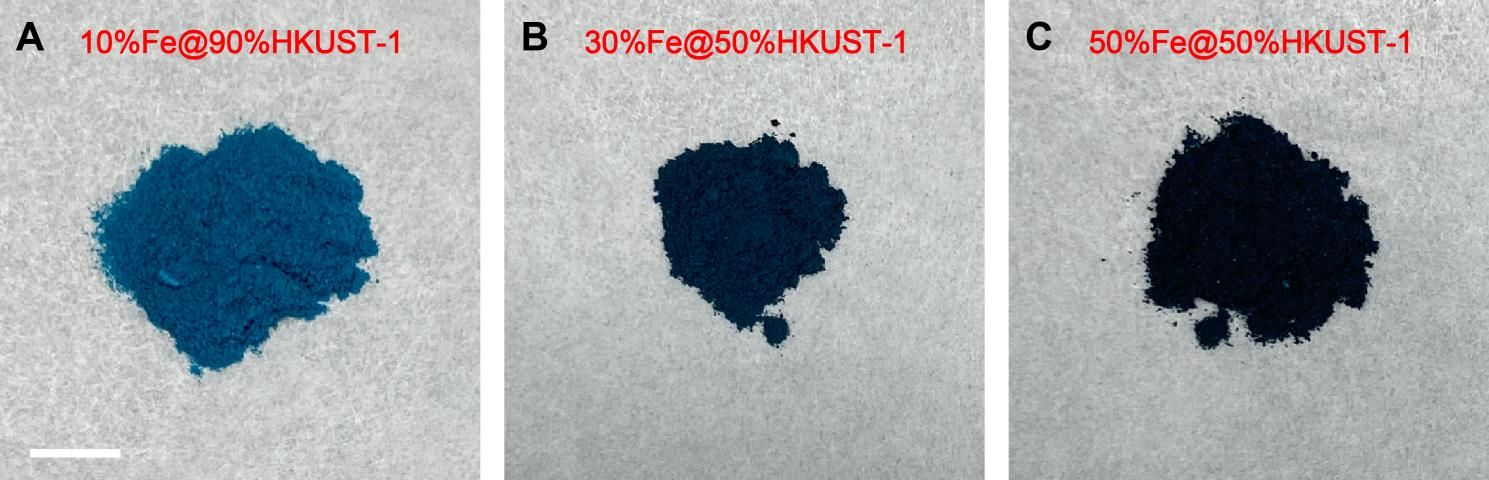


**Figure S11.** The optical image of (A) 10%Fe@90%HKUST-1, (B) 30%Fe@70%HKUST-1, and (C) 50%Fe@50%HKUST-1. Scale bars are (A-C) 2 cm.


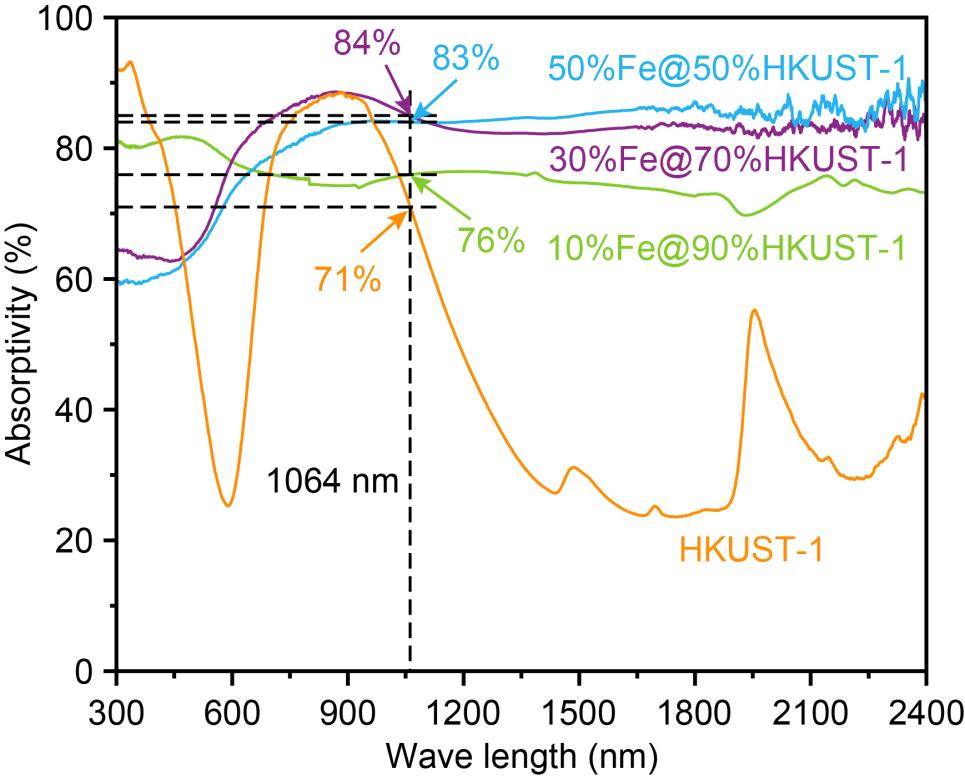


**Figure S12.** Experimental light absorption profiles of HKUST-1, 10%Fe@90%HKUST-1, 30%Fe@70%HKUST-1 and 50%Fe@50%HKUST-1.


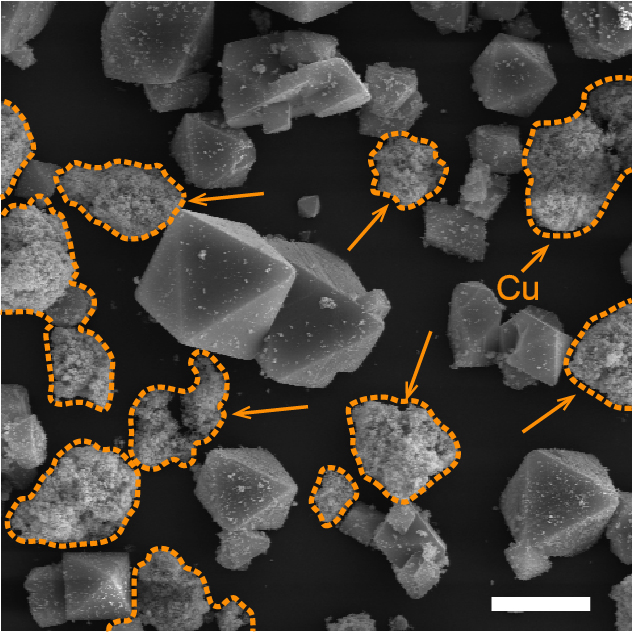


**Figure S13.** SEM images of 10%Fe@90%HKUST-1 by physical mixing. Scale bars are 20 μm.


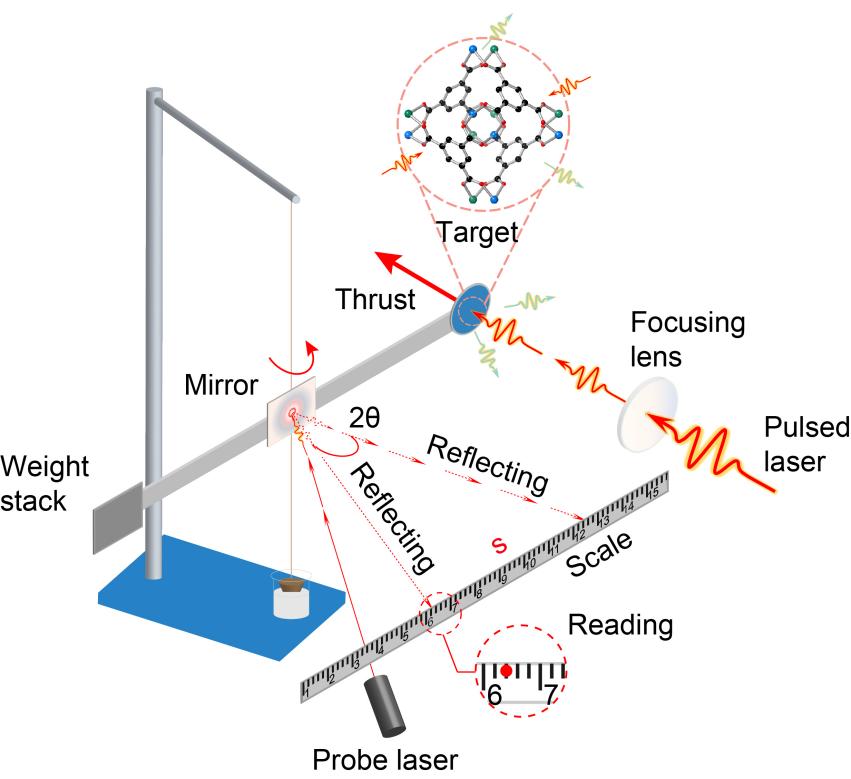


**Figure S14.** Schematic representations of the experimental arrangement used to measure the impulse in pulsed laser processing.


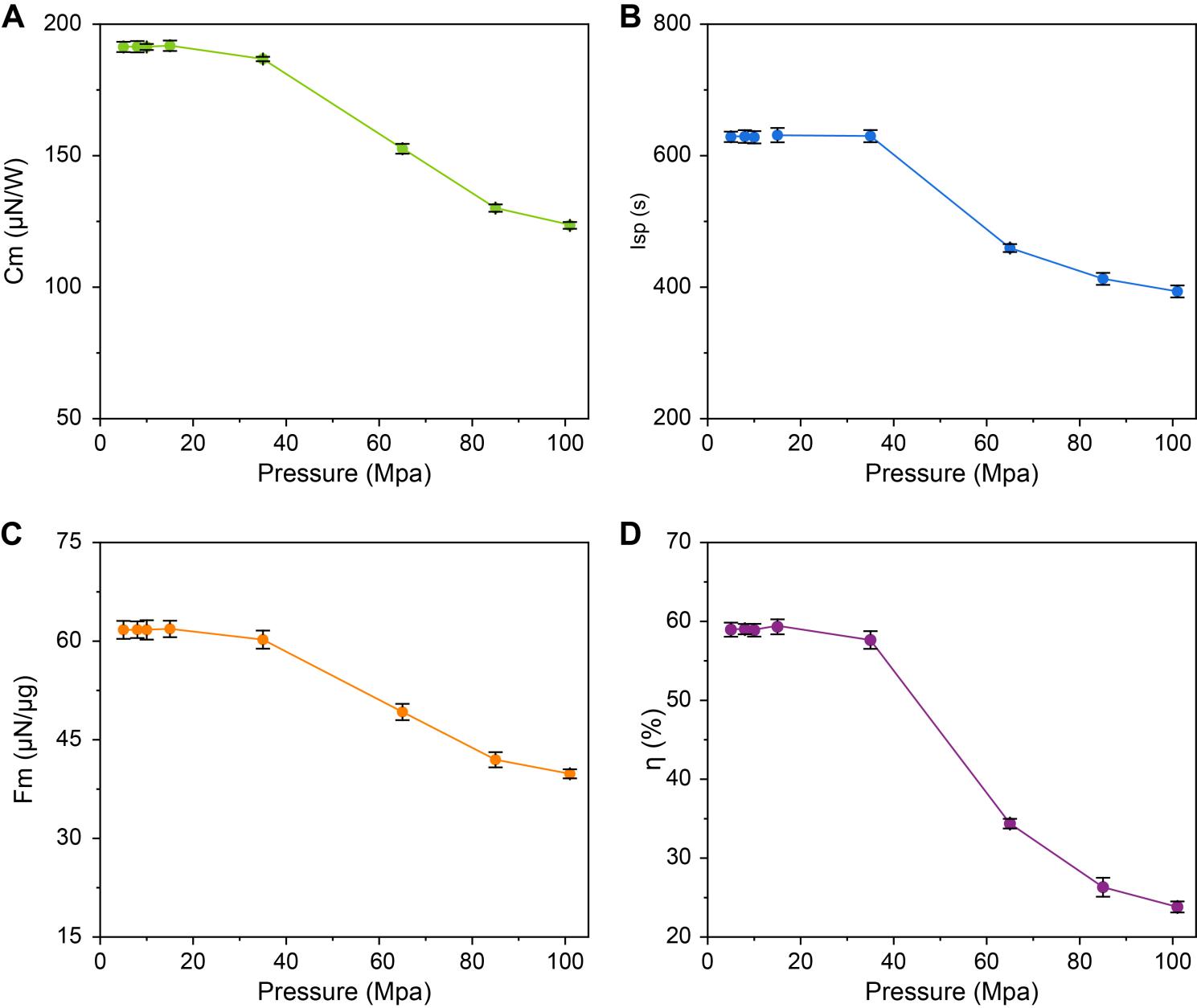


**Figure S15.** PLMP performance of FeCu-MOF-S under different ambient pressure.


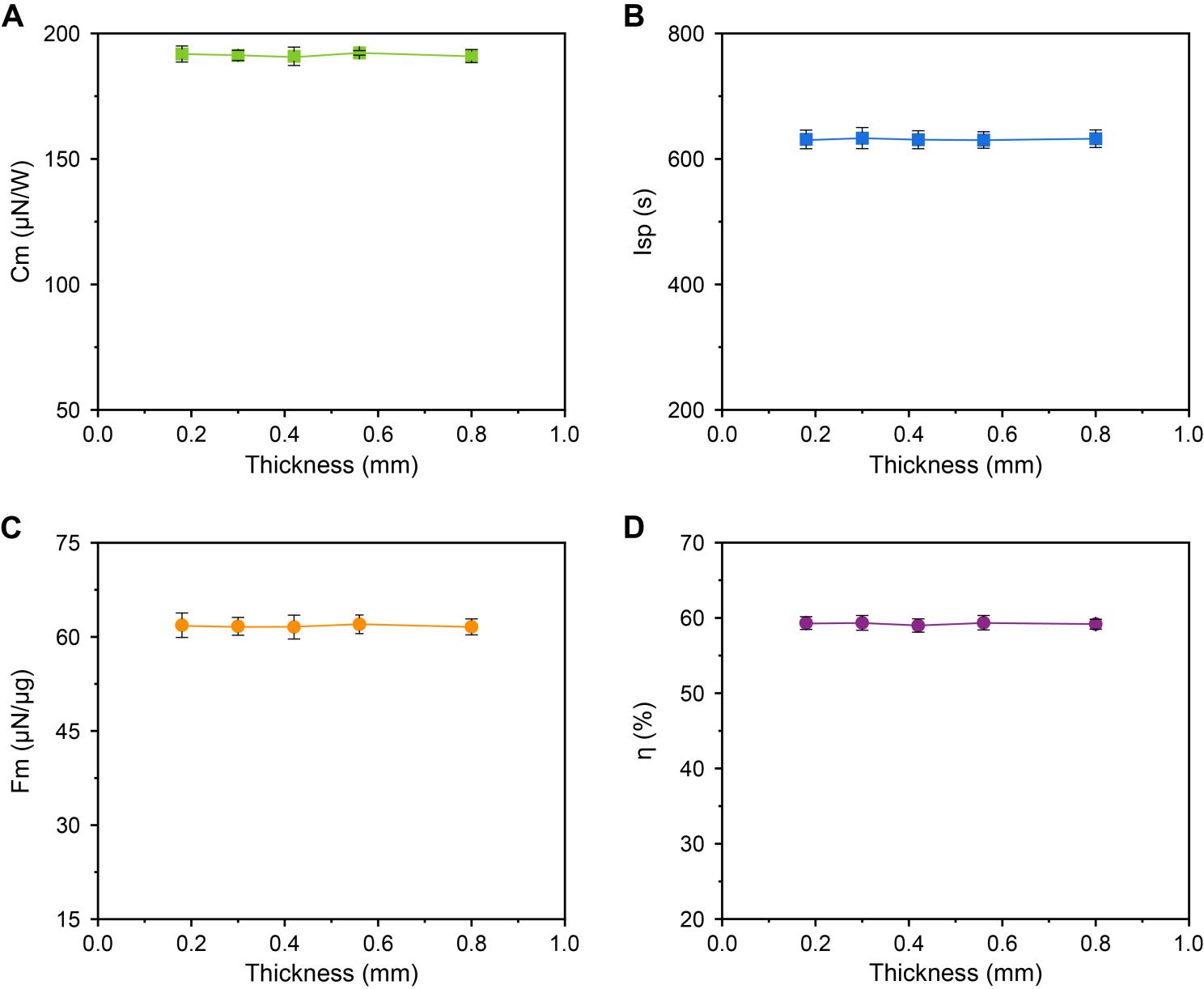


**Figure S16.** PLMP performance of FeCu-MOF-S under different thickness of sheets.


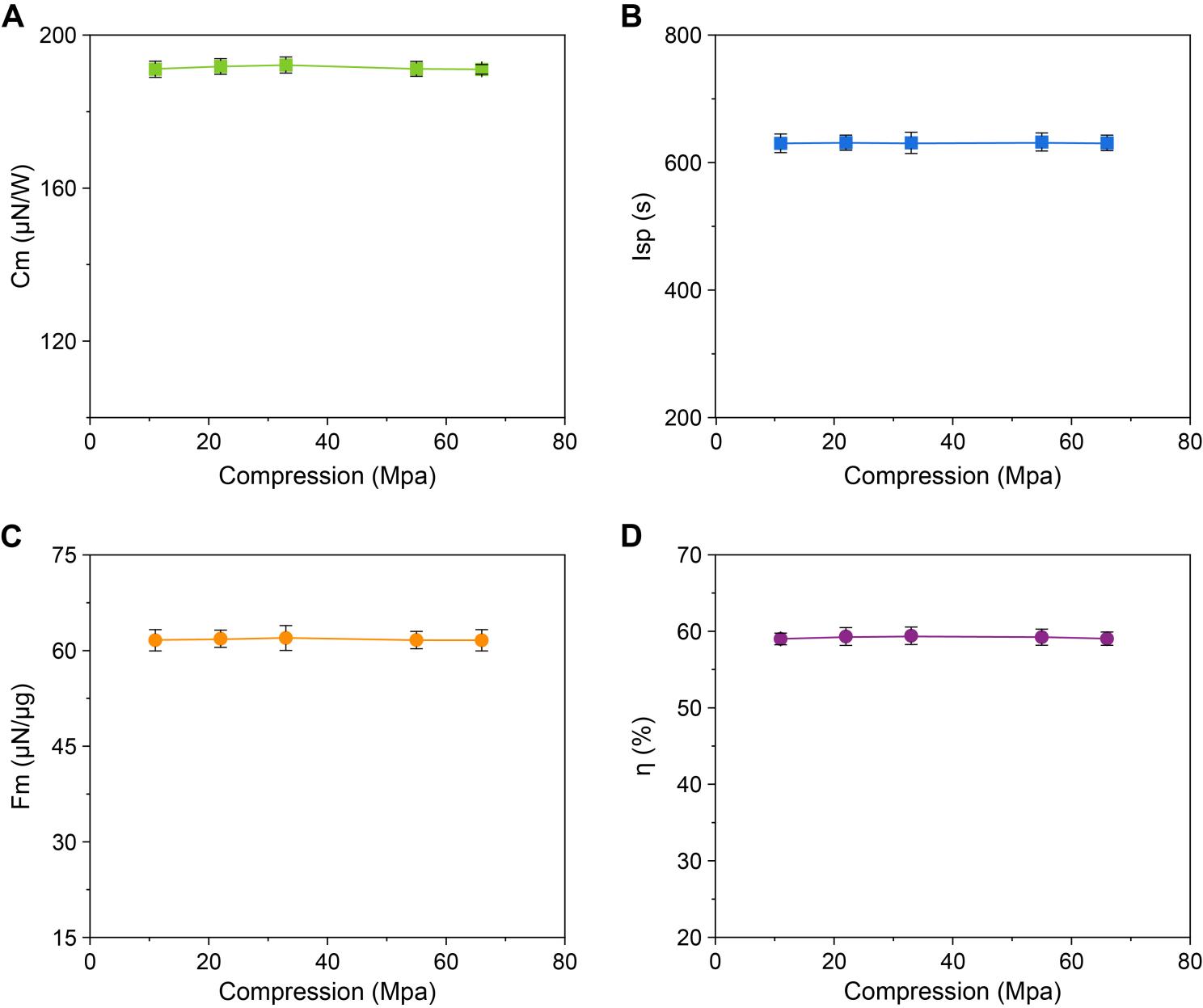


**Figure S17.** PLMP performance of FeCu-MOF-S under different compression pressure.


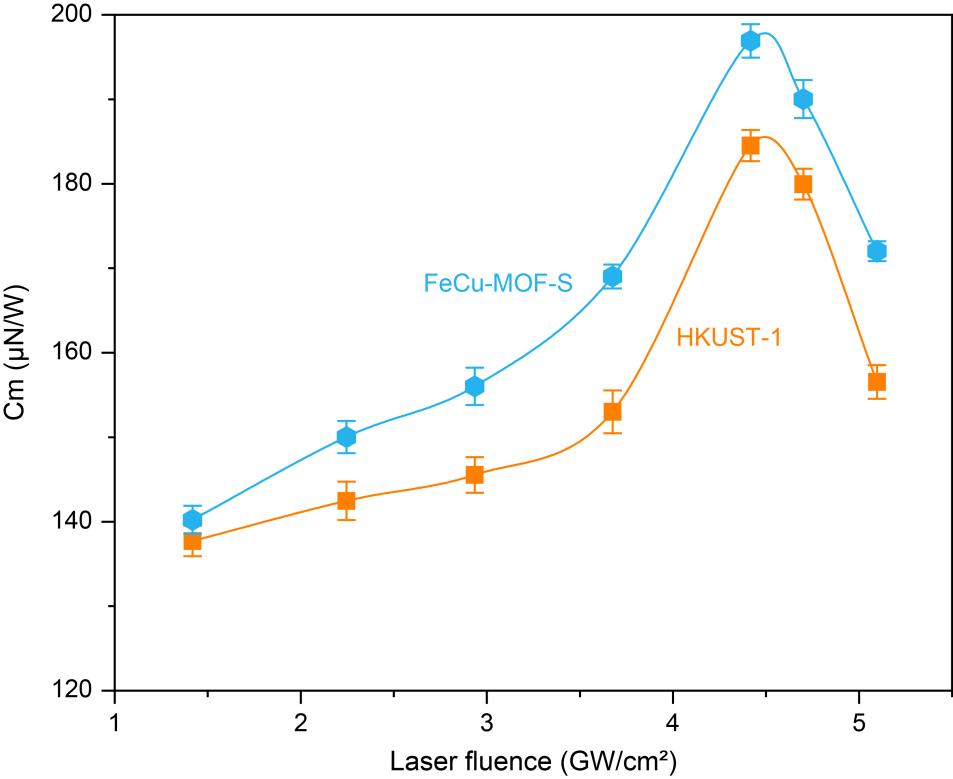


**Figure S18.** Impulse coupling coefficients of FeCu-MOF-S and HKUST-1 under different laser fluence.

.


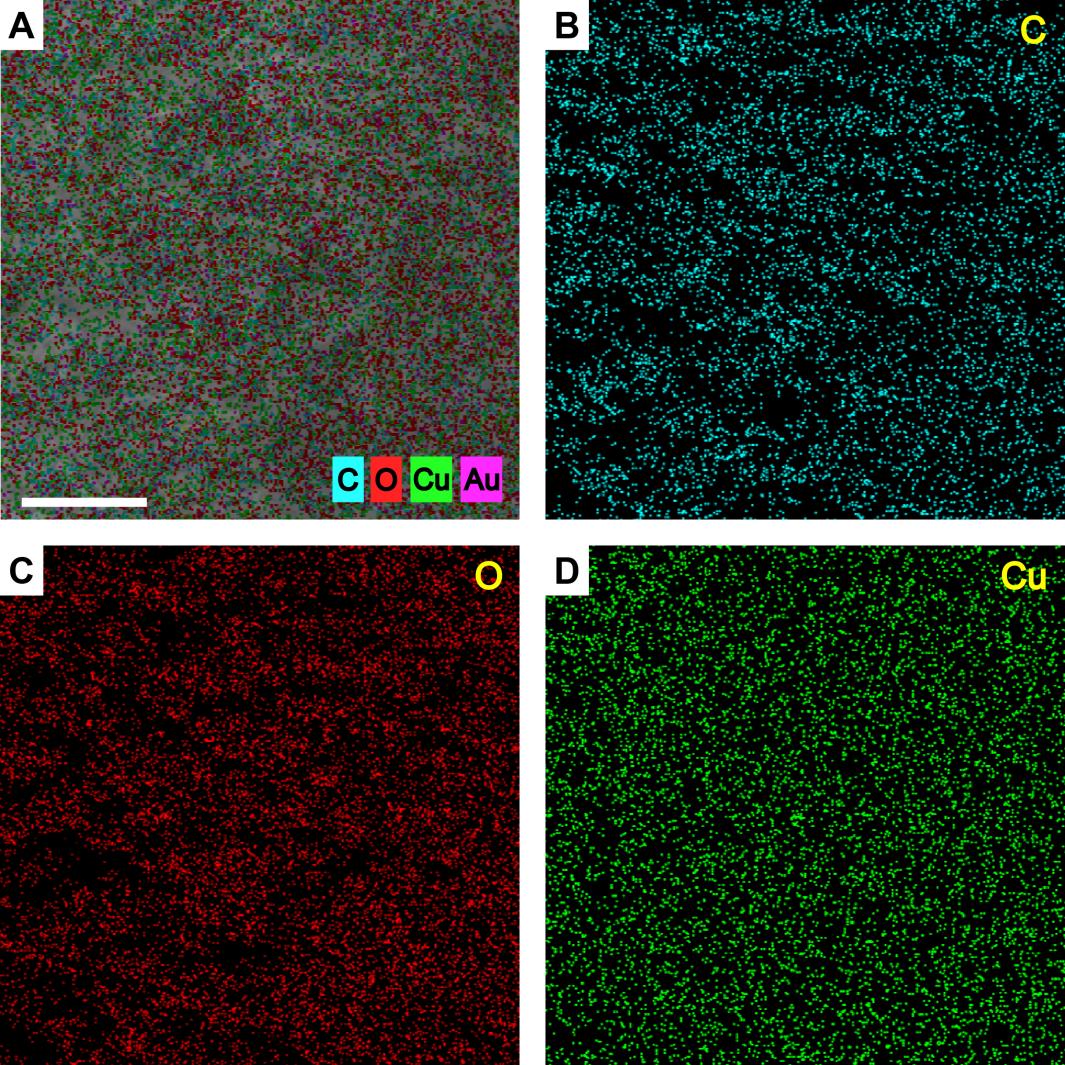


**Figure S19.** Elemental distributions mappings of HKUST-1 after laser ablation. Scale bars are 50 μm. The Au element is produced by the gold spray process.


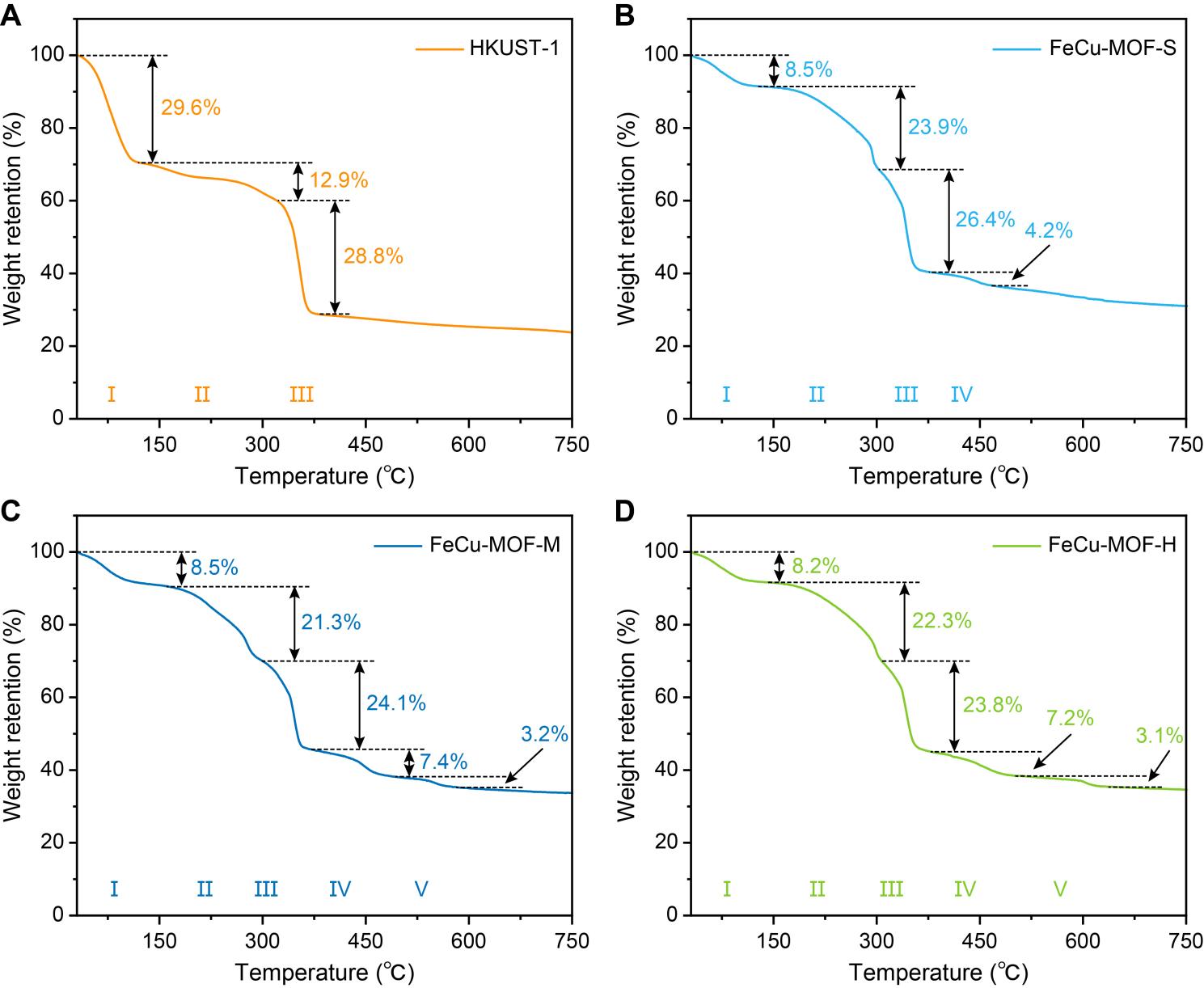


**Figure S20.** TGA curves of (A) HKUST-1, (B) FeCu-MOF-S, (C) FeCu-MOF-M and (D) FeCu-MOF-H.


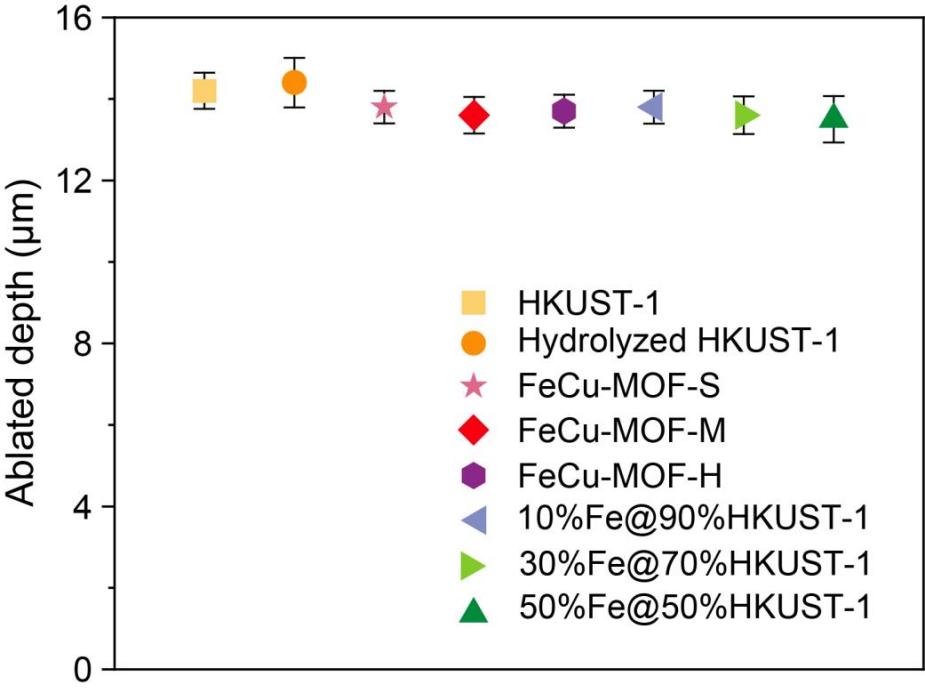


**Figure S21.** The single-pulse ablation depth of different MOFs.


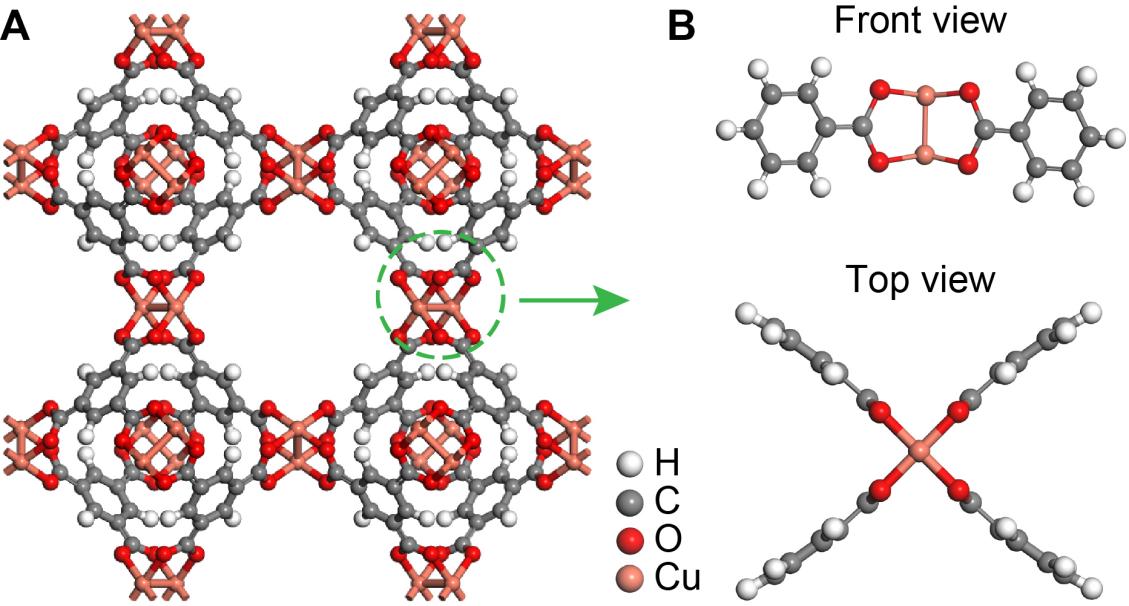


**Figure S22.** DFT-calculated models of (A) the crystal unit cell structure of HKUST-1 and (B) front and top views of the simplified model.


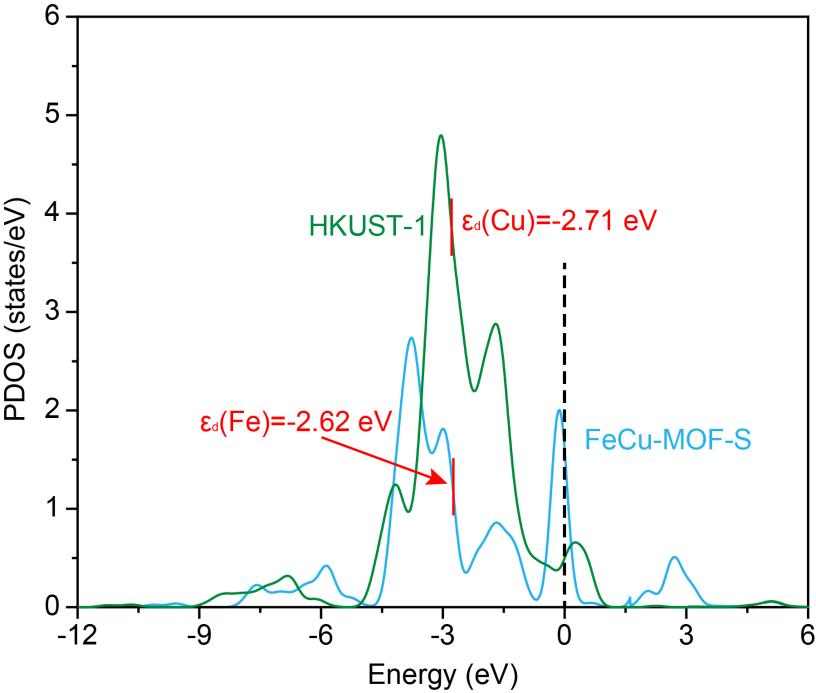


**Figure S23.** PDOS and *d*-band center of Cu-3*d* in HKUST-1 and Fe-3*d* in CuFe-MOF-S. Th*e d*-band center of the Fe atom in CuFe-MOF-S is higher than that of the Cu atom in HKUST-1.


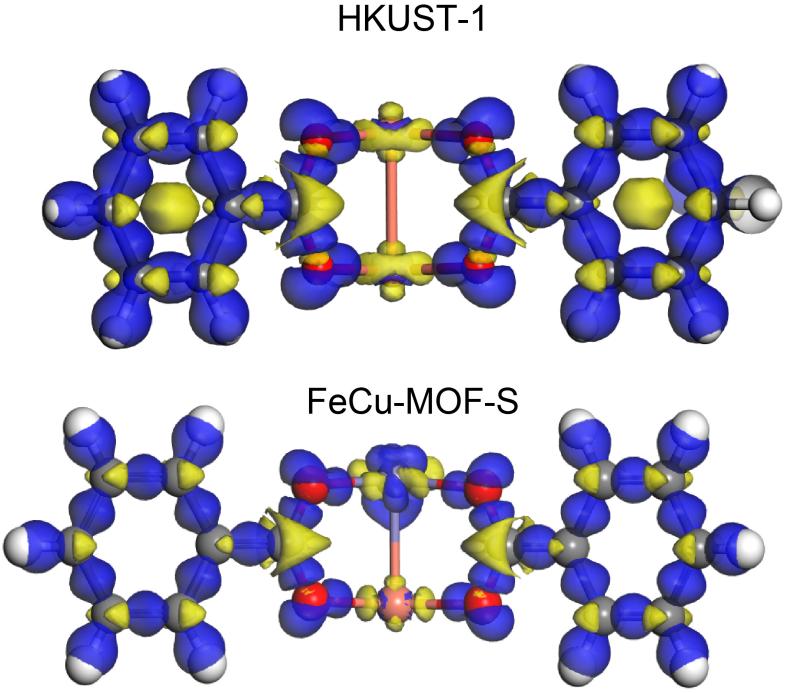


**Figure S24.** Charge density difference of HKUST-1 and FeCu-MOF-S.


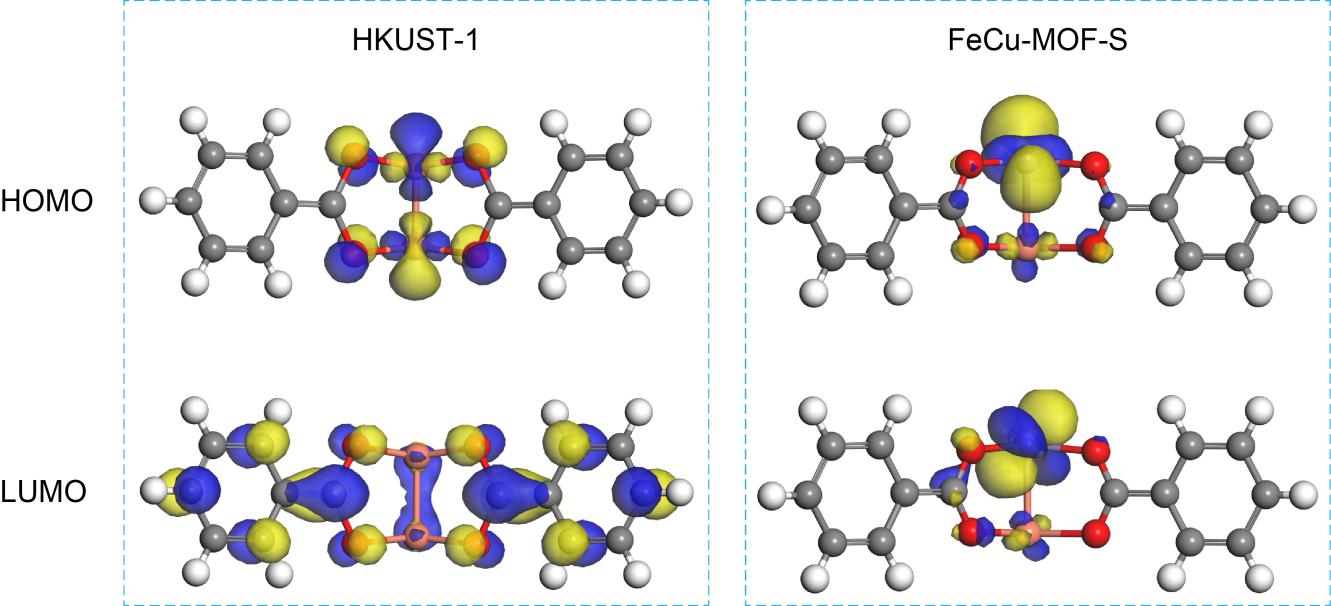


**Figure S25.** HOMO and LUMO distributions in HKUST-1 and FeCu-MOF-S.


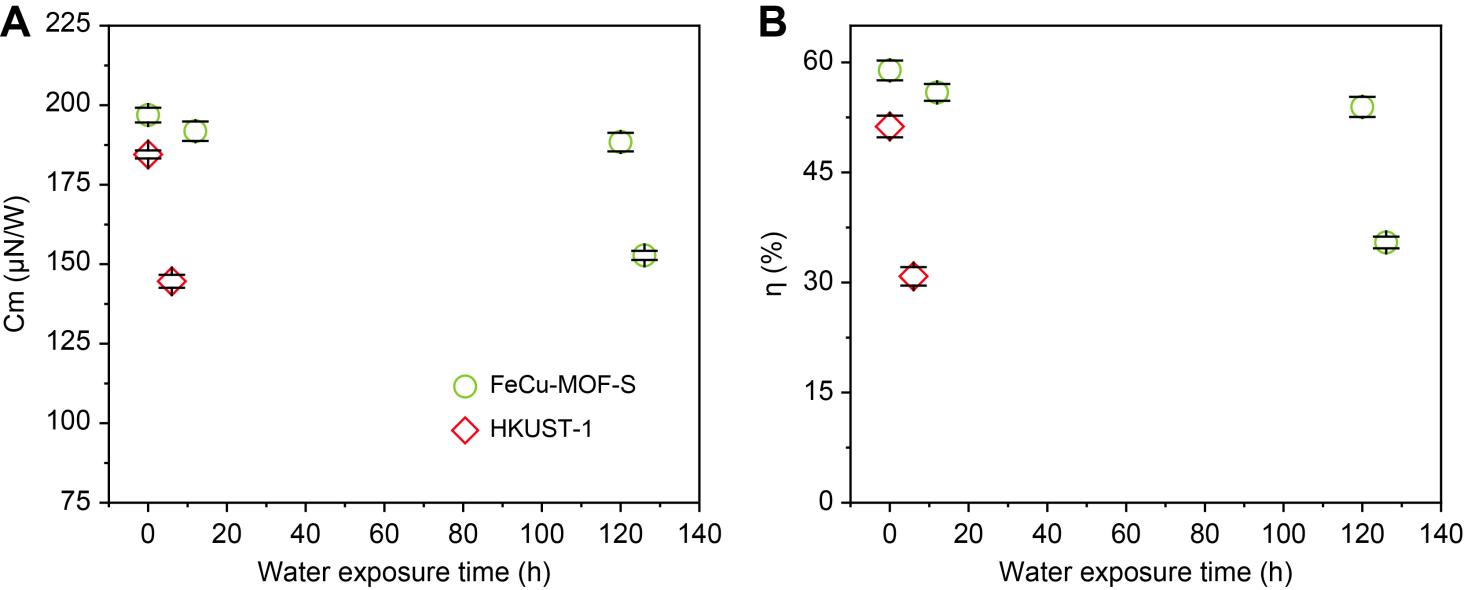


**Figure S26.** Performance comparison of FeCu-MOF-S and HKUST-1 before and after water exposure: (A) The impulse coupling coefficients, and (B) The PLMP efficiencies.

**Table S1.** The concentrations of Fe and Cu in FeCu-MOFs were measured by SEM-EDS.

| **Items** | Materials | Cu  (atomic%) | Fe  (atomic%) | Fe:Cu | Cu  (weight%) | Fe  (weight%) | Fe:Cu |
| --- | --- | --- | --- | --- | --- | --- | --- |
| 1 | FeCu-MOF-S | 13.93 | 0.71 | 1:19.6 | 21.93 | 1.07 | 1:20.5 |
| 2 | FeCu-MOF-M | 10.47 | 1.07 | 1:9.8 | 20.75 | 1.77 | 1:11.7 |
| 3 | FeCu-MOF-H | 11.46 | 2.55 | 1:4.5 | 20.4 | 3.78 | 1:5.4 |

**Table S2.** The concentrations of Fe and Cu in FeCu-MOFs were measured by ICP-OES.

| **Items** | Materials | Cu (ppma) | Fe (ppma) | Fe:Cu |
| --- | --- | --- | --- | --- |
| 1 | FeCu-MOF-S | 220.5 | 10.9 | 1:20.2 |
| 2 | FeCu-MOF-M | 193.6 | 18.8 | 1:10.3 |
| 3 | FeCu-MOF-H | 166.3 | 35.8 | 1:4.7 |

**Table S3.** Textural parameters of different materials.

| Samples | BET (m^2^/g) | Total pore volume (cm^3^/g) |
| --- | --- | --- |
| HKUST-1 | 1552 | 0.58 |
| FeCu-MOF-S | 1468 | 0.76 |
| FeCu-MOF-M | 1480 | 0.82 |
| FeCu-MOF-H | 1256 | 0.74 |

**Table S4.** Density of different materials by using the same compression pressure.

| Materials | Density (g/cm^3^) | Materials | Density (g/cm^3^) |
| --- | --- | --- | --- |
| HKUST-1 | 1.312 | Fe | 6.822 |
| FeCu-MOF-S | 1.319 | 10%Fe/90%MOF | 1.469 |
| FeCu-MOF-M | 1.261 | 30%Fe/70%MOF | 1.776 |
| FeCu-MOF-H | 1.252 | 50%Fe/50%MOF | 2.057 |

**Table S5.** The performance comparison of different propellants comparison with previous works.

| **Items** | **Materials** | ***C*_m_ (μN/W)** | ***I*_sp_ (s)** | ***η* (%)** | **Years** | **ref.** |
| --- | --- | --- | --- | --- | --- | --- |
| 1 | FeCu-MOF-M | 191.8 | 631 | 59.3 | 2025 | This work |
| 2 | HKUST-1 | 183.7 | 568 | 51.15 | 2024 | ref. 2 |
| 3 | HKUST-1derived Graphene-Metal MetaMaterial  (GMM-(HKUST-1)) | 97.4 | 1072.9 | 51.22 | 2024 | ref. 20 |
| 4 | MOF derived Carbon-encapsulated-Nano-Metal Composite (CNMC-450) | 89.3 | 969.6 | 42.4 | 2024 | ref. 13 |
| 5 | Triazene polymer (TP) | 60 | 110 | 3.2 | 2002 | ref. 33 |
| 6 | TP | 60 | 200 | 5.9 | 2003 | ref. 34 |
| 7 | Polytetrafluoroethylene (PTFE) | 58 | 960 | 27.3 | 2011 | ref. 12 |
| 8 | Polyvinyl chloride (PVC) | 67.9 | 1064 | 35.4 | 2009 | ref. 35 |
| 9 | PVC | 70 | 200 | 6.9 | 2003 | ref. 36 |
| 10 | PVC | 77.3 | 208.6 | 7.9 | 2004 | ref. 37 |
| 11 | PVC | 162 | 151 | 12.0 | 2007 | ref. 38 |
| 12 | Poly(lactic acid)+Poly(1,12- dodecamethylene 2,5-furandicarboxylate) (PDoF)+reduced graphene oxide (rGO) | 139 | 309 | 21 | 2025 | ref. 4 |
| 13 | 5% Graphene +95% PTFE | 16.2 | 1012 | 8.2 | 2022 | ref. 3 |
| 14 | 10% TiO_2_ + 90% PTFE | 4.74 | 9648.8 | 22.4 | 2021 | ref. 11 |
| 15 | Polyimide (PI) | 5.1 | 365 | 0.9 | 2011 | ref. 39 |
| 16 | Al+PVC | 16 | 125 | 1.0 | 2011 | ref. 40 |
| 17 | Triazene polymer (TP) | 34 | 186 | 3.1 | 2002 | ref. 41 |
| 18 | TP+C | 35 | 190 | 3.3 | 2002 | ref. 42 |
| 19 | Polymethyl methacrylate (PMMA) + C | 37 | 900 | 16.3 | 2004 | ref. 43 |
| 20 | Polychlorotrifluoroethylene (PCTFE) | 55 | 164 | 4.4 | 2008 | ref. 44 |
| 21 | PVC+C | 120 | 650 | 38.2 | 2004 | ref. 45 |
| 22 | Polyvinyl nitrate (PVN) +C | 120 | 655 | 38.5 | 2004 | ref. 46 |
| 23 | PVN +C | 310 | 137 | 20.8 | 2007 | ref. 47 |
| 24 | Polyoxymethylene (POM) +C | 310 | 150 | 22.8 | 2008 | ref. 48 |

**Supplemental References**

S1. H. Yu, H. Li, Y. Wang, L. Cui, S. Liu, J. Yang. Brief review on pulse laser propulsion. *Optics and Laser Technology*, 2018, 100, 57-74.

S2. C. Yu, W. Zhou, H. Chang, Y. Chen. Experimental research on impulse coupling characteristics and plasma plume dynamics of a nanosecond pulsed laser irradiated aluminum target. IEEE Access, 2020, 8, 205272-205281.

S3. Z. Zheng, T. Liang, S. Zhang, L. Gao, H. Gao, Z. Zhang. Ablation of carbon-doped liquid propellant in laser plasma propulsion. Applied Physics A, 2016, 122 (4), 1-4.

S4. Z. Y. Zheng, H. Gao, L. Gao, J. Xing, Z. J. Fan, A. G. Dong, Z. L. Zhang, Laser plasma propulsion generation in nanosecond pulse laser interaction with polyimide film, Applied Physics A 115(4) (2014), 1439-1443.

S5. D. Zhang, J. Wu, R. Zhang, H. Zhang, Z. He. High precision micro-impulse measurements for micro-thrusters based on torsional pendulum and sympathetic resonance techniques. Review of Scientific Instrument, 2013, 84(12),125113.

S6. L. Jiao, J. Cai, H. Ma, G. Li, L. Li, Z. Shen, Z. Tang. Research on applications of rectangular beam in micro laser propulsion. Applied Surface Science, 2014, 301, 481-487.

S7. D. Förster, S. Faas, R. Weber, T. Graf. Thrust enhancement and propellant conservation for laser propulsion using ultra-short double pulses, Applied Surface Science, 2020, 510, 145391.

S8. M. Keidar, T. Zhuang, A. Shashurin, G. Teel, D. Chiu, J. Lukas, S. Haque, L. Brieda, C. Fusion. Electric propulsion for small satellites, Plasma Physics and Controlled Fusion, 2015, 57, 1-10.

S9. Y. Jamil, H. Saeed, M. Ahmad, S. Khan, H. Farooq, M. Shahid, K. Zia, N. Amin. Measurement of ablative laser propulsion parameters for aluminum, Co–Ni ferrite and polyurethane polymer, Applied Physics A, 2012, 110(1), 207-210.

S10. C. Luke, Diode laser-driven microthrusters: A new departure for micropropulsion. AIAA Journal, 2002, 40, 310-318.
